# Supplementary material for: SQM2.20: Semiempirical quantum-mechanical scoring function yields DFT-quality protein–ligand binding affinity predictions in minutes
Source: Nat Commun. 2024 Feb 6;15:1127. doi: 10.1038/s41467-024-45431-8 (PMC10847445; doi:10.1038/s41467-024-45431-8)
Supplement: Supplementary file 1 — Supplementary Information [file 41467_2024_45431_MOESM1_ESM.pdf]

Supplementary Information

SQM2.20: Semiempirical quantum-mechanical scoring function yields DFT-quality protein–ligand binding affinity predictions in minutes

Adam Pecina,[1+] Jindřich Fanfrlík,[1+] Martin Lepšík[1] & Jan Řezáč[1*]

[1] *Institute of Organic Chemistry and Biochemistry of the Czech Academy of Sciences*
Flemingovo náměstí 2
166 10, Prague
Czech Republic

[+] These authors contributed equally to this work.

E-mail: rezac@uochb.cas.cz

Index

Index 1
S1) PL-REX dataset description 2
S2) SQM scoring results 5
S3) Alternative SQM Scoring Protocols 6
S4) PM6-D3H4X vs. DFT 7
S5) Effect of the P–L geometries on the score 8
S6) SQM2.20 calculations on the whole protein 10
S7) Effect of the protein structure selection 11
S8) Relationship between SQM2.20 and absolute binding free energy 12
S9) List of crystal structures used 13
S10) Plots of all scoring functions 15
S11) 2D structures of the ligands 42
S12) References 61

S1) PL-REX dataset description

Supplementary Note 1. *Selection rules.*

The ten targets that make up the PL-REX dataset are listed in this section. In addition to a brief introduction to each of them, we provide the reference to the source data and add our notes that are important for working with the system. The PL-REX dataset uses all ligands from the original publications referenced here if they meet two criteria:

1) The structure of the P-L complex is available from experiment, or it can be easily modeled based on an experimental structure with a very similar ligand. We have only added small functional groups that fit into the binding sites without clashes, have a small number of rotatable bonds (i.e. a small number of possible conformations) and retain the core of the parent ligand.

2) Unambiguous experimental affinity is available, and was measured consistently with the other ligands in the series. We did not consider ligands for which the affinity is not well defined, such as weakly-binding ones with estimated activities (e.g. reported as IC_{50} > 100 μM). No ligands were discarded arbitrarily, even if they were problematic for scoring.

Supplementary Note 2. *Main characteristics of PL-REX systems.*

01-CA2

Target: Human carbonic anhydrase II (CA2) is a zinc metalloenzyme. It is essential for maintaining general acid-base equilibrium.
Source: Reference 1.
PDB codes: 5NXG, 5NXI, 5NXO, 5NXP, 5NXV, 5NXW, 5NY1, 5NY3, 5NY6, and 5NYA
Notes: The amide group which interacts with the Zn^{2+} is ionized (-NH^{-}). The formal charge on the ionized nitrogen atoms was set to -1 for use in MOZYME.

02-HIV-PR

Target: HIV-1 protease (PR) is a dimeric aspartic protease, target of anti-HIV drugs.
Source: References 2-4.
PDB codes: 1HSG, 1IZH, 2Q54, 3EKX, 3EL1, 3OXC, 1HXW, 1T3R, 2Q5K, 2Q55, 3NU3, and 5HVP
Notes: Consistently measured K_i values of HIV-1 protease inhibitors from Konvalinka lab[2,3] and Schiffer lab[4] were used, taking advantage of the fact that five clinically used inhibitors (SQV, RTV, APV, LPV and ATV) were measured in both laboratories and showed no systematic shift with only minor deviations within the experimental uncertainties.[5] For these five inhibitors, the K_i values from Konvalinka lab[2,3] were used. Two ligands (OE and SQ) from Ref. 3 were not used because their central NH_2^+ group would require a different protonation of the catalytic aspartate dyad (unprotonated) than for the other inhibitors. Ten compounds with 5S stereochemistry from Ref. 4 were built from the crystal structures of *13e* or *14a* complexes (2Q54 or 2Q55 PDB codes, respectively).
The OD1 "lower" oxygen of Asp25' of HIV-1 protease was considered protonated. Wat301 bridging two carbonyl/sulfonyl oxygens of the ligands was retained. The ligand of the 5HVP complex (acetyl-pepstatin) changes its protonation state upon binding (proton transfer to the COO^{-} group of the inhibitor).

03-CK2

Target: Protein kinase formerly known as casein kinase 2 (CK2) is a pleiotropic Ser/Thr protein kinase with hundreds of downstream targets, which have a variety of cellular functions. It is important in gene-expression regulation, the synthesis and degradation of proteins, as well as the signaling and suppression of apoptosis and it serves as a pharmacological target for treatment of different neoplastic diseases. Here, we use *Zea mays* CK2 protein kinase.
Source: 16 CK2 X-ray crystal structures from L. A. Pinna laboratory, with consistently measured K_i values and with the X-ray crystal structures available.[6-11]

PDB codes: 1F0Q, 1J91, 1M2P, 1M2Q, 1M2R, 1ZOE, 1ZOG, 1ZOH, 2OXD, 2OXX, 2OXY, 3KXG, 3KXH, 3KXM, 3KXN, and 3PVG.
Notes: The 3PVG changes its protonation state upon binding (proton transfer to the COO^- group of the inhibitor).

04-AR

Target: Human Aldose reductase (AR) catalyzes the conversion of glucose to sorbitol. It has been established as a target for treatment of diabetic complications.
Source: References 12-16.
PDB codes: 1US0, 2IKG, 2IKH, 2IKI, 2IKJ, 4LAZ, 4LB4, 4LBS, 4XZH, 4LAU, 4LB3, 4LBR, 4QXI, and 4XZI.
Notes: Dataset of 7 inhibitors with crystal structures and K_i values from ITC measurements extended by datasets of eight compounds with crystal structures and IC_{50} values. All the IC_{50} values were scaled according to the IDD388 inhibitor, which is present in both series. The resulting dataset consisted of 14 inhibitors. Cofactor (NADP) was retained and considered as part of the protein.

05-Cath-D

Target: Cathepsin D (Cath-D) is a pepsin-family aspartic protease. Its primary role is lysosomal digestion of proteins. Cath-D is implicated in numerous pathologies including several neurodegenerative conditions, osteoarthritis and acute pancreatitis.
Source: Reference 17.
PDB codes: 6QBG, 6QBH, and 6QCB.
Notes: Original dataset of 35 macrocyclic inhibitors with IC_{50} values, 3 of them with an X-ray crystal structure. We use 10 systems which cover the range of IC_{50} values from 0.9 to 2 000 nM, and either have the crystal structure or can be built from the crystal structure with confidence. One aspartate (Asp231) of the catalytic dyad was protonated on OD2 (the "upper" oxygen). The alternative B conformation of Met309 was used.

06-BACE1

Target: Beta secretase 1, BACE1 is the major β-secretase for the generation of amyloid-β peptides in neurons and a potential target related to Alzheimer's disease.
Source: D3R grand challenge 4.[18]
PDB codes: 5QCO, 5QCP, 5QCR, 5QCT, 5QCU, 5QCV, 5QCX, 5QCY, 5QCZ, 5QD0, 5QD1, 5QD2, 5QD3, 5QD5, 5QD9, and 5QDA.
Notes: The catalytic aspartate Asp228 was protonated on OD1. Wat523 was retained.

07-JAK1

Target: JAK1 is a human tyrosine kinase essential for signaling for certain type I and type II cytokines. JAK1 plays a critical role in initiating responses to multiple major cytokine receptor families.
Source: References 19-24.
PDB codes: 4EHZ, 4EI4, 4E4L, 4E4N, 4E5W, 4FK6, 4IVB, 4IVC, 4IVD, 4I5C, 4K6Z, and 4K77.
Notes: Six water molecules were retained as a part of the protein, specifically Wat1302, Wat1329, Wat1349, Wat1351, Wat1419 and Wat1456 in 4IVD.

08-Trypsin

Target: Trypsin is a serine protease, found in the digestive system of many vertebrates. Trypsin inhibitors interfere with digestion activity and have an antinutritional effect.
Source: References 25-29.
PDB codes: 1K1I, 1K1J, 1K1L, 1K1M, 1K1N, 2ZHD, 2ZQ2, 3lJJ, 3LJO, 5MNG, 5MO2, 6SY3, 6T0M, 6T0P, and 6T5W.
Notes: The protonation of ligands was assessed according to the literature. It has been shown that approximately one proton is transferred from the buffer to the ligand of the 1K1I complex (to the COO^- group) upon complex formation, whereas about "0.5 proton" is released upon binding of the ligand of the 1K1L complex (from the

NH_2^+ group).[25] Additionally, a proton is also transferred from the buffer to the ligands of the 6SY3, 6T0M, 6T0P, 6T3Q, 6T5W and 6TS3 complexes (to the pyridine groups).[28] Catalytic residues Ser195 and His56 are charged.[30] Ten water molecules were considered, specifically Wat1005, Wat1006, Wat1017, Wat1024, Wat1025, Wat1040, Wat1093, Wat1137 and Wat1124 in 1K1I and Wat396 in 1K1O.

09-CDK2

Target: Cyclin-dependent kinase 2 (CDK2) is the most studied member of the CDK family of Ser/Thr protein kinases. It is an essential component of cell-cycle regulation.
Source: All ligands with IC_{50} values from Reference 31.
PDB codes: 3QQK, 3QTR, 3QTU, 3QTX, 3QU0, 3RAH, 3RAL, 3RKB, 3RK7, 3RMF, 3RPV, 3R8U, 3R8Z, 3R9N, 3S0O, 3S1H, 3QTQ, 3QTS, 3QTW, 3QTZ, 3QXP, 3RAK, 3RJC, 3RK5, 3RK9, 3RNI, 3RPY, 3R8V, 3R9D, 3SQQ, and 3S00.
Notes: Residues Tyr15, Phe146, Gly147 and Leu148 were optimized by AMBER ff19SB force field with IGB7 implicit solvent model (see Methods) in order to remove the clashes of Tyr15:OH with Gly147:O and Arg126:NH2 with Leu148:N.

10-MMP12

Target: Matrix metalloproteinase-12 (MMP12) may play a role in aneurysm formation and studies in mice and humans suggest a role in the development of emphysema.
Source: References 32-34.
PDB codes: 3EHX, 3EHY, 3F15, 3F16, 3F17, 3F18, 3F19, 3F1A, 3LK8, 3N2U, 3N2V, 3NX7, 3RTS, 3RTT, 4GUY, 5LAB, 6RD0, and 6RLY.
Notes: Two ligands, i.e. of the 3EHX and 3EHY complexes, change their protonation state upon binding (proton transfer to the COO^- group of the inhibitor).

S2) SQM scoring results

Supplementary Figure 1. *SQM2.20 scores plotted against the binding free energies (ΔG_{bind}^0) derived from the experimental affinities for each target series of the PL-REX dataset. Analogous plots for the other scoring functions discussed in the paper are provided in the Section 10 of the Supplementary Information. All energies are in kcal/mol. Source data are provided as a Source Data file.*

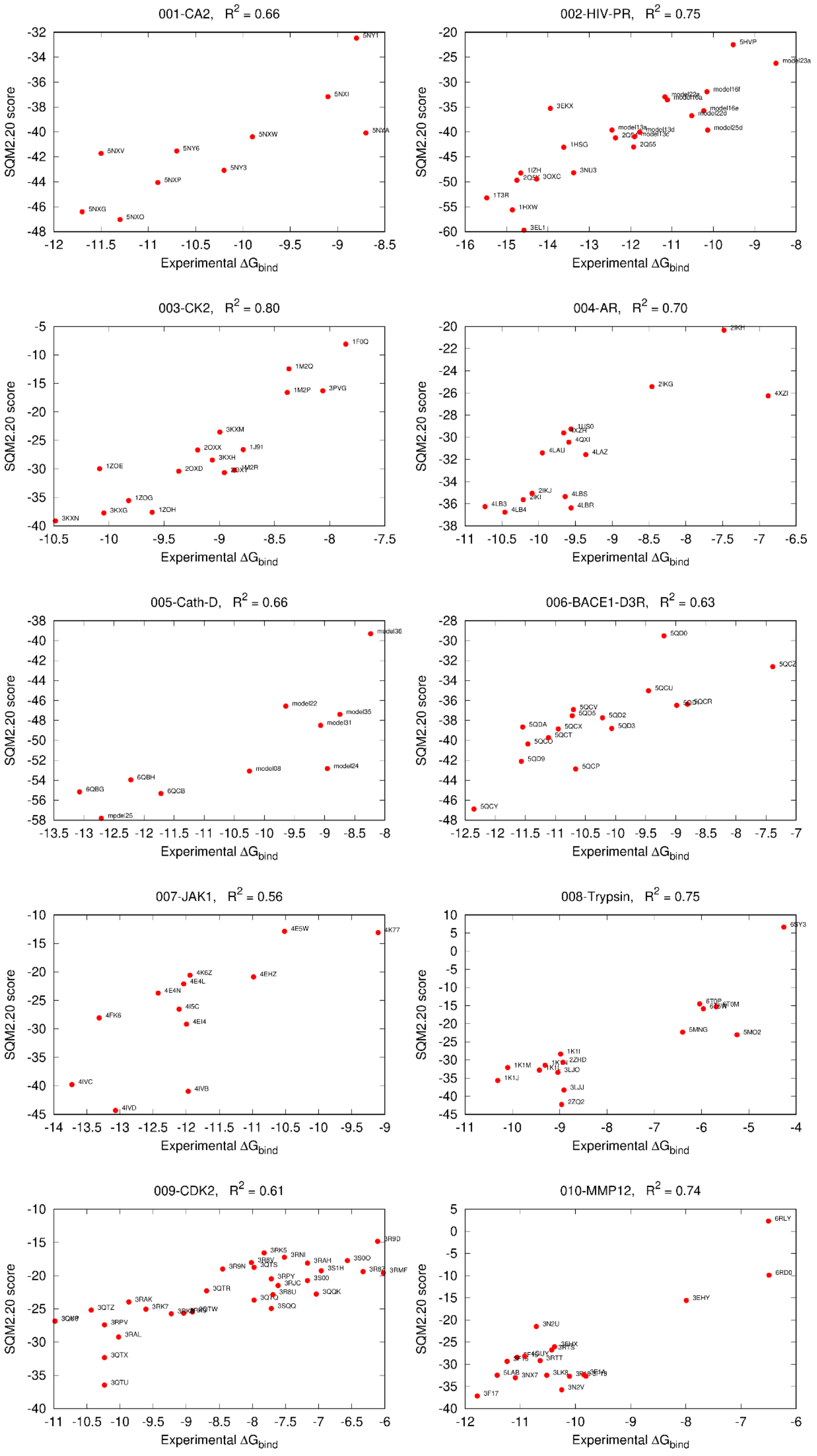

S3) Alternative SQM Scoring Protocols

Supplementary Note 3. *SQM-Score performance.*

The performance of SQM2.20 was evaluated by correlating the scores to the binding free energies (ΔG_{bind}^0) derived from the experimental affinities, and quantified by the squared Pearson coefficient, R^2 (Supplementary Figure 1). The performance of SQM2.20 score was compared to its derivatives based on PM7 and default PM6-D3H4X corrections with COSMO solvent model (Tab. S1). Plots of these scores are also available in the Section 10 of the Supplementary Information.
As the benchmark for the PM6-D3H4X method used in the SQM2.20 score, we used the state-of-the-art density functional theory (DFT) using a range-separated hybrid ωB97X-D3BJ functional / DZVP-DFT basis set. Their correlation for the leading term of the SQM2.20 score, the gas-phase interaction energy, ΔE_{int}, is shown in Supplementary Figure 2.

Supplementary Table 1. *Squared Pearson coefficient, R^2, of the correlation between the SQM2.20 score and its PM7/COSMO and default PM6-D3H4X/COSMO derivatives and the experimental binding free energies, ΔG_{bind}^0, calculated on the PL-REX dataset. Source data are provided as a Source Data file.*

**Target**	**SQM2.20**	**PM7/COSMO**	**default PM6-D3H4X/COSMO**
01-CA2	0.67	0.70	0.41
02-HIV-PR	0.75	0.63	0.77
03-CK2	0.81	0.27	0.27
04-AR	0.70	0.64	0.68
05-Cath-D	0.66	0.71	0.53
06-BACE1	0.63	0.67	0.40
07-JAK1	0.56	0.71	0.52
08-Trypsin	0.75	0.86	0.72
09-CDK2	0.61	0.41	0.50
10-MMP12	0.74	0.20	0.76
**Average**	**0.69**	**0.58**	**0.56**

S4) PM6-D3H4X vs. DFT

Supplementary Note 4. *Timing of benchmark calculations.*

Here we compare the timing of the SQM and DFT calculations discussed in the paper. It has to be emphasized that we are comparing the timing of just the calculation of ΔE_{int} to which DFT was applied. The timing of the complete SQM2.20 scoring protocol is discussed separately in Section S6 of the Supplementary Information.

Supplementary Table 2. *Average CPU time in hours (normalized to 1 CPU) for ΔE_{int} calculation for PL-REX datasets as calculated using DFT (on AMD 7H12, 2.6 GHz CPU) compared to CPU time in minutes using PM6-D3H4X (Intel Xeon Gold 6140, 2.30 GHz). n.d. not determined because the DFT calculations failed to converge for three complexes with iodine-containing ligands in the 04-AR target.*

**Target**	**Trimmed model (~1,000 atoms)**		**Default model (~2,000 atoms)**
	**DFT**	**PM6-D3H4X**	**PM6-D3H4X**
01-CA2	537.7 hrs	1.8 min	8.4 min
02-HIV-PR	749.3 hrs	2.4 min	7.2 min
03-CK2	560.2 hrs	6.6 min	9.0 min
04-AR	n.d.	6.0 min	12.0 min
05-Cath-D	686.7 hrs	1.8 min	7.8 min
06-BACE1	758.9 hrs	3.0 min	9.6 min
07-JAK1	715.3 hrs	2.4 min	6.0 min
08-Trypsin	517.5 hrs	3.0 min	7.2 min
09-CDK2	739.8 hrs	2.4 min	5.4 min
10-MMP12	525.7 hrs	1.8 min	4.8 min
**Average**	**643.5 hrs***	**3.1 min**	**7.7 min**
**Minimum**	**517.5 hrs***	**1.8 min**	**4.8 min**
**Maximum**	**758.9 hrs***	**6.6 min**	**12.0 min**

* *04-AR target excluded from the statistics*

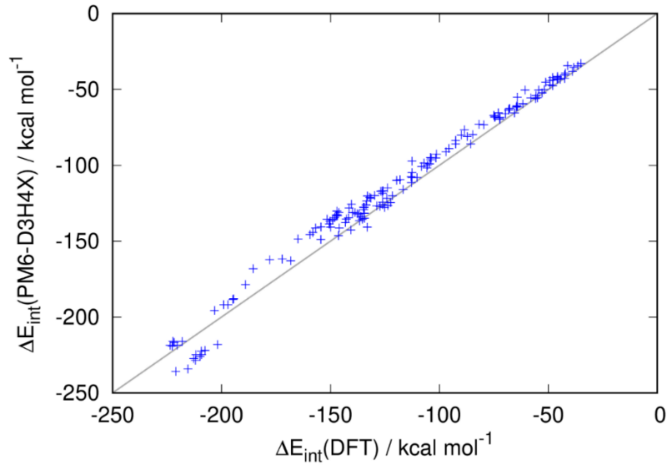

Supplementary Figure 2. *Correlation, R^2, of interaction energies in the gas phase calculated at PM6-D3H4X and DFT levels (see Methods) over the PL-REX dataset on PL-REX geometries. Source data are provided as a Source Data file.*

S5) Effect of the P–L geometries on the score

Supplementary Note 5. *Performance of SFs on MM-optimized geometries.*

We also tested MM-optimized geometries for scoring by standard SF and ML methods, which resulted in only small deterioration in correlations as compared to the PL-REX geometries (Supplementary Figure 3; Supplementary Table 3). It is evident that the performance of standard SFs is not significantly improved by replacing MM-optimized geometries with PL-REX geometries, contrary to the SQM2.20 which benefits from geometries of higher quality.

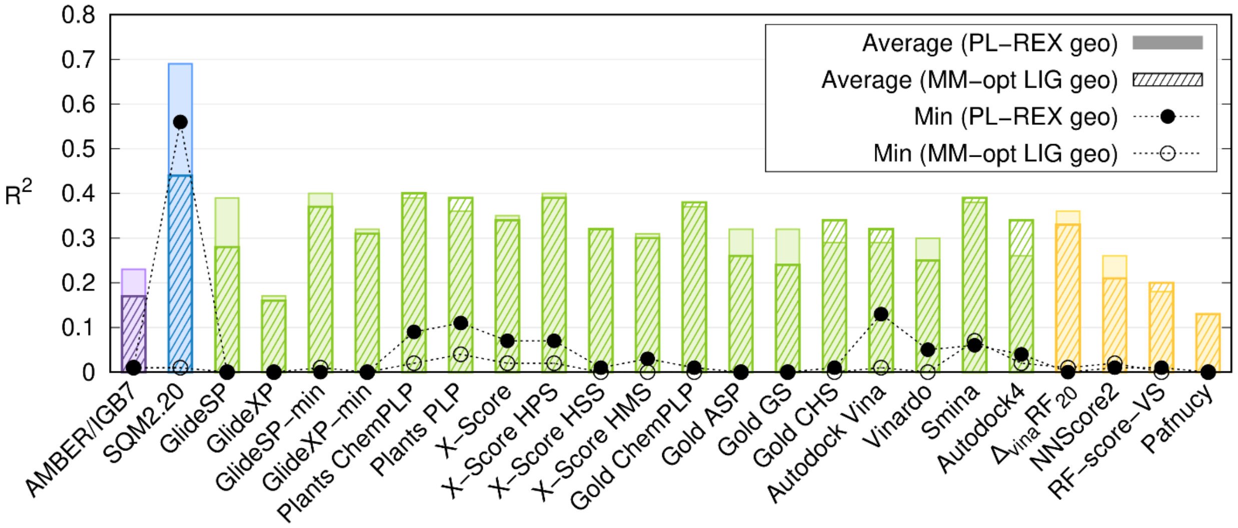

Supplementary Figure 3. *Average and minimal correlation, R^2, of MM score (purple), SQM2.20 (blue), standard scoring functions (green) and ML methods (orange) over the PL-REX dataset on MM-opt LIG geometries (hatched) and PL-REX geometries (solid, light). Source data are provided as a Source Data file.*

Supplementary Table 3. *Correlation, R^2, of SQM2.20 and other scoring functions with the experimental binding free energies on **A.** PL-REX geometries and **B.** MM-optimized ligands (heavy atoms of the protein frozen), i.e. MM-opt LIG geometries. Values with one asterisk (*) indicate no correlations obtained with Glide Scores greater than 10,000. Values with two asterisks (**) indicate a series where calculations for a few ligands failed (two ligands of 02-HIV-PR, one ligand of 03-CK2 and one ligand in 10-MMP12) and thus the correlation was calculated for >90% of ligands. The scale of R^2 goes from red (0.00), via yellow (0.50) to green (1.00). Source data for individual scoring functions are provided as a Source Data files.*

A.

on PL-REX geo	SQM2.20	GlideSP	GlideXP	GlideSP -min	GlideXP -min	Plants ChemPLP	Plants PLP	X-Score	X-Score HPS	X-Score HSS	X-Score HMS	Gold ChemPLP	Gold ASP
01-CA2	0.67	0.00*	0.00*	0.00	0.24	0.20	0.26	0.07	0.14	0.07	0.03	0.19	0.34
02-HIV-PR	0.75	0.12	0.01	0.08	0.01	0.22	0.14	0.13	0.07	0.01	0.34	0.01	0.00
03-CK2	0.81	0.08	0.08	0.04	0.00	0.14	0.15	0.12	0.38	0.10	0.06	0.23**	0.34**
04-AR	0.70	0.57	0.67	0.59	0.52	0.57	0.49	0.74	0.76	0.77	0.51	0.70	0.19
05-Cath-D	0.66	0.61	0.09	0.76	0.55	0.80	0.72	0.56	0.62	0.41	0.58	0.61	0.45
06-BACE1	0.63	0.60	0.38	0.67	0.72	0.39	0.38	0.76	0.64	0.74	0.70	0.31	0.34
07-JAK1	0.56	0.74	0.23	0.73	0.53	0.62	0.62	0.32	0.52	0.19	0.25	0.75	0.47
08-Trypsin	0.75	0.76	0.02	0.84	0.30	0.68	0.51	0.48	0.60	0.66	0.26	0.58	0.85
09-CDK2	0.61	0.25	0.06	0.22	0.20	0.09	0.11	0.13	0.13	0.07	0.18	0.16	0.22
10-MMP12	0.74	0.21	0.22	0.10	0.10	0.17	0.20	0.18	0.17	0.16	0.20	0.14	0.00
AVERAGE	0.69	0.39	0.17	0.40	0.32	0.39	0.36	0.35	0.40	0.32	0.31	0.37	0.32
Minimal	0.56	0.00	0.00	0.00	0.00	0.09	0.11	0.07	0.07	0.01	0.03	0.01	0.00

on PL-REX geo	Gold GS	Gold CHS	Autodock Vina	vinardo	smina	Autodock4	ΔvinaRF20	NNScore2	RF-score-VS	pafnucy	av. ± st.dev. standard SFs	
01-CA2	0.07	0.07	0.13	0.05	0.06	0.06	0.30	0.27	0.10	0.01	0.12	0.11
02-HIV-PR	0.36	0.09	0.15	0.21**	0.13	0.10	0.58	0.01	0.01	0.05	0.13	0.14
03-CK2	0.00**	0.01**	0.30	0.27	0.28	0.04	0.01	0.01	0.47	0.01	0.14	0.14
04-AR	0.84	0.61	0.53	0.61	0.68	0.47	0.56	0.11	0.01	0.18	0.53	0.22
05-Cath-D	0.43	0.57	0.57	0.48	0.64	0.34	0.60	0.62	0.01	0.21	0.51	0.20
06-BACE1	0.26	0.43	0.19	0.21	0.59	0.38	0.02	0.09	0.19	0.24	0.42	0.23
07-JAK1	0.77	0.58	0.19	0.35	0.55	0.20	0.72	0.55	0.07	0.37	0.47	0.22
08-Trypsin	0.19	0.29	0.49	0.44	0.65	0.57	0.52	0.30	0.67	0.20	0.49	0.22
09-CDK2	0.20	0.21	0.15	0.19	0.11	0.16	0.24	0.12	0.02	0.05	0.15	0.06
10-MMP12	0.06	0.04	0.26	0.17	0.10	0.27	0.00	0.57	0.26	0.00	0.16	0.12
AVERAGE	0.32	0.29	0.29	0.30	0.38	0.26	0.36	0.26	0.18	0.13		
Minimal	0.00	0.01	0.13	0.05	0.06	0.04	0.00	0.01	0.01	0.00		

B.

on MM-opt LIG geo	SQM2.20	GlideSP	GlideXP	GlideSP -min	GlideXP -min	Plants ChemPLP	Plants PLP	X-Score	X-Score HPS	X-Score HSS	X-Score HMS	Gold ChemPLP	Gold ASP
01-CA2	0.08	0.00*	0.00*	0.01	0.39	0.18	0.22	0.10	0.23	0.12	0.03	0.00	0.00
02-HIV-PR	0.42	0.08	0.00	0.08	0.00	0.28	0.19	0.05	0.02	0.00	0.20	0.03	0.00
03-CK2	0.71	0.14	0.03	0.33	0.04	0.02	0.04	0.02	0.27	0.00	0.00	0.02**	0.11**
04-AR	0.57	0.39	0.34	0.36	0.59	0.83	0.75	0.81	0.72	0.80	0.62	0.84	0.35
05-Cath-D	0.53	0.52	0.12	0.69	0.37	0.76	0.73	0.53	0.53	0.34	0.59	0.69	0.33
06-BACE1	0.01	0.30	0.12	0.33	0.39	0.19	0.16	0.74	0.60	0.75	0.71	0.24	0.07
07-JAK1	0.57	0.46	0.28	0.60	0.45	0.63	0.59	0.31	0.49	0.18	0.26	0.67	0.61
08-Trypsin	0.65	0.54	0.65	0.76	0.61	0.79	0.89	0.59	0.71	0.78	0.33	0.86	0.93
09-CDK2	0.26	0.30	0.00	0.38	0.24	0.09	0.10	0.12	0.13	0.07	0.16	0.17	0.18
10-MMP12	0.58	0.11**	0.11**	0.15	0.03	0.24	0.25	0.16	0.17	0.18	0.12	0.29	0.01
AVERAGE	0.44	0.28	0.16	0.37	0.31	0.40	0.39	0.34	0.39	0.32	0.30	0.38	0.26
Minimal	0.01	0.00	0.00	0.01	0.00	0.02	0.04	0.02	0.02	0.00	0.00	0.00	0.00

on MM-opt LIG geo	Gold GS	Gold CHS	Autodock Vina	vinardo	smina	Autodock4	ΔvinaRF20	NNScore2	RF-score-VS	pafnucy	av. ± st.dev. standard SFs	
01-CA2	0.00	0.00	0.29	0.21	0.08	0.02	0.19	0.05	0.34	0.00	0.11	0.12
02-HIV-PR	0.20	0.01	0.10	0.10**	0.07	0.10	0.17	0.06	0.05	0.01	0.08	0.08
03-CK2	0.16**	0.37**	0.11	0.01	0.13	0.05	0.26	0.03	0.51	0.00	0.12	0.14
04-AR	0.34	0.70	0.47	0.24	0.81	0.76	0.51	0.02	0.07	0.18	0.52	0.26
05-Cath-D	0.47	0.37	0.42	0.25	0.55	0.51	0.45	0.38	0.06	0.11	0.44	0.20
06-BACE1	0.20	0.40	0.01	0.00	0.60	0.43	0.08	0.17	0.23	0.36	0.32	0.23
07-JAK1	0.43	0.57	0.52	0.39	0.69	0.51	0.75	0.69	0.08	0.26	0.47	0.18
08-Trypsin	0.52	0.62	0.72	0.70	0.71	0.62	0.66	0.67	0.65	0.19	0.66	0.17
09-CDK2	0.00	0.14	0.14	0.17	0.14	0.21	0.23	0.03	0.00	0.20	0.14	0.10
10-MMP12	0.08	0.18	0.38	0.47	0.09	0.16	0.01	0.04	0.00	0.01	0.15	0.12
AVERAGE	0.24	0.34	0.32	0.25	0.39	0.34	0.33	0.21	0.20	0.13		
Minimal	0.00	0.00	0.01	0.00	0.07	0.02	0.01	0.02	0.00	0.00		

S6) SQM2.20 calculations on the whole protein

Supplementary Note 6. *Model size verification.*

In this section, we compare the averaged correlations between the SQM.2.20 scores and the experimental binding free energies derived from experimental affinities, using the default model and the whole protein. It has to be emphasized that we are comparing the results (correlations and timing) of the complete SQM2.20 scores calculated as a sum of terms defined in the *Eq. 1* of the paper. In addition to this, we discuss the timing of ΔE_{int} term calculation at PM6-D3H4X and DFT level separately, in the section S4 of the Supplementary Information.

Here, we had verified that the default model, i.e. using all residues within 10 Å around all the overlaid ligands in each target protein, perfectly reproduces the computationally more demanding scoring in the whole protein. The results and timing of the SQM2.20 score calculations (on one CPU) averaged for each PL-REX target is shown in Supplementary Table 4. The timing for each complex using the default model is depicted in Supplementary Figure 4.

Supplementary Table 4*. Averaged correlations, R^2, and computational time in minutes, on the 10 protein-ligand systems of the PL-REX data set, using 10 Å cutoff of the systems (the default model) and using the whole protein in the QM part.*

	**Default model (from 1,270 to 2,199 atoms)**		**Whole protein (from 2,413 to 5,841 atoms)**	
**Target**	**SQM2.20**	**Averaged timing (in minutes)**	**SQM2.20 (full protein)**	**Averaged timing (in minutes)**
01-CA2	0.67	16.5	0.67	54.5
02-HIV-PR	0.75	17.3	0.70	51.0
03-CK2	0.81	18.3	0.81	126.0
04-AR	0.70	22.9	0.71	118.3
05-Cath-D	0.66	17.3	0.66	86.4
06-BACE1	0.63	36.7	0.65	105.4
07-JAK1	0.56	27.2	0.57	69.1
08-Trypsin	0.75	21.6	0.76	39.5
09-CDK2	0.61	14.7	0.61	80.2
10-MMP12	0.74	10.4	0.75	26.2
**Average**	**0.69**	**20.3**	**0.69**	**75.7**
**Minimal**	**0.56**	**10.4**	**0.57**	**26.2**

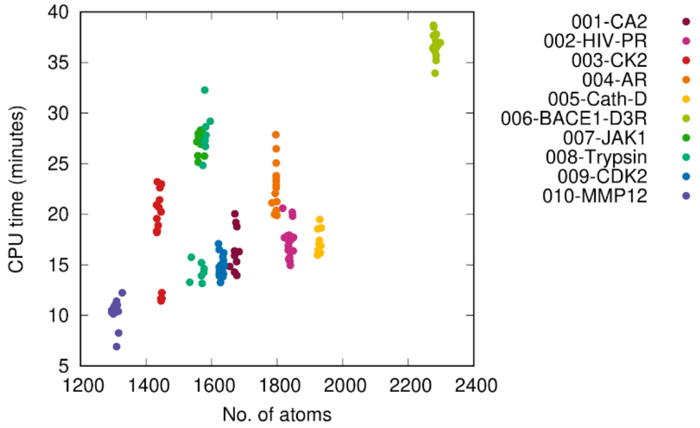

Supplementary Figure 4*. The timing of the SQM2.20 score calculation (on one CPU) for each complex of the PL-REX data set using the default model. Source data are provided as a Source Data file.*

S7) Effect of the protein structure selection

Supplementary Note 7. *SQM2.20 results on different protein structures.*

In this section, we analyze how the choice of receptor conformation affects the results of the SQM2.20 scoring, comparing the default protein structure selection protocol with two alternatives. The default PL-REX structures have been selected so that the protein that can best accommodate all the ligands (i.e., the protein with the largest measured closest distance between the protein and all the ligands) and is labelled “least contact” here. In the first alternative, we consider the protein with the second largest closest contact distance obtained in the same way ("2nd least contact") as a check of the sensitivity of the results to smaller variations in the protein structure. In the second alternative, we apply a completely different but also rational rule to select the native protein of that ligand which is the most similar to all the other ligands (i.e., has the highest average Tanimoto coefficient with all the other ligands). This alternative is referred to as the "most similar ligand".

The results are summarized in Supplementary Table 5. It is clear that the choice of the structure which provides enough space for the ligands is important for the success of the scoring. In case that the second best structure is used, the average correlation drops to R^2 of 0.56, which is mainly due to the poor results in 06-BACE1, 07-JAK1 and 09-CDK2. The second best structures are generally similar to the best ones but there are important differences in the binding sites. In the case of 06-BACE1, the conformation of the Thr72 - Gln73 - Gly74 loop differs. In 07-JAK1, Arg1007 of the second best structure collides with the largest ligand of the set. The second best structure of 09-CDK2 varies in the conformation of the Asn132 side chain. These three systems are also problematic when the native protein of the most similar ligand is used. Again, the average R^2 drops to 0.56.

Finally, each ligand can be scored in its own crystal structure. We do not use this approach because the scoring function neglects some terms that may cancel out if a single protein structure is used for all the ligands. Also, preparing multiple crystals for the calculations and ensuring that they are as consistent as possible is a tedious task. As a demonstration, we performed such an analysis for the 01-CA2 target where each ligand was scored in its own crystal. The computed scores were slightly more favorable than when using a single receptor structure, on average by 0.7 kcal/mol. However, the obtained correlation R^2 of 0.43 was significantly lower than that when using a single receptor structure where R^2 ranged from 0.52 to 0.67 (Supplementary Table 5). This result highlights the importance of using a single representative protein structure when an end-point scoring function of this type is used.

Supplementary Table 5. *Representative protein conformations selected according to different criteria and respective correlations of SQM2.20 with the experimental affinities. The crystals used in PL-REX are in bold. Source data are provided as a Source Data files.*

	Crystal used			Squared Pearson coefficient (R^2)		
Target	Least contact	2nd least contact	Most similar ligand	Least contact	2nd least contact	Most similar ligand
01-CA2	**5NXG**	5NXP	5NXP	**0.67**	0.52	0.52
02-HIV-PR	**2AQU**	3OXC	2Q54	**0.75**	0.77	0.73
03-CK2	**3KXN**	1ZOE	**3KXN**	**0.81**	0.71	**0.81**
04-AR	**4XZH**	4QXI	2IKI	**0.70**	0.61	0.73
05-Cath-D	6QBH	6QBG	**6QCB**	0.46	0.53	**0.66**
06-BACE1	**5QCZ**	5QD3	5QCO	**0.63**	0.28	0.22
07-JAK1	**4IVD**	4EHZ	4EI4	**0.56**	0.38	0.42
08-Trypsin	**1K1I**	6T0M	2ZQ2	**0.75**	0.73	0.65
09-CDK2	**3R9H**	3R9D	3RAK	**0.61**	0.42	0.14
10-MMP12	**3EHY**	3N2U	3NX7	**0.74**	0.66	0.72
Average				0.67	0.56	0.56

S8) Relationship between SQM2.20 and absolute binding free energy

Supplementary Note 8. *Comparison between the scores and the experimental affinities.*

Although the SQM2.20 score calculates the key terms of the binding free energy using accurate computational methods, the result is not on the same scale as the experimental ΔG_{bind}^0. The neglect of some terms that would weaken the binding, most importantly of the thermal effects and the dynamics of the system, makes the score in the energy units larger. This is a common feature of simplified end-point scoring functions, and some of them address it by an empirical scaling of the final values of the score. We have chosen not to use such a scaling in order to keep the SQM2.20 score free of any system-specific parameters. This limitation is mitigated by working only with the correlation between the scores and the experimental free energies, as we do throughout this work. To quantify this effect, we applied a linear regression to the ten series of ΔG_{bind}^0 and SQM2.20 scores, and we list the resulting slopes in Supplementary Table 6. As expected, the slope values are greater than 1, ranging from 2.2 to 5.7 in all the systems except 03-CK2 with a slope of 11.1, but with an excellent correlation ($R^2 = 0.80$).
A second effect that prevents a direct comparison between the scores and the experimental affinities is a shift of the scale, again due to the neglect of some terms of the binding free energy. This shift can be considered as a constant which characterizes each target and its ligand series and can be removed by subtracting the average score of the series and adding the average ΔG_{bind}^0. The scores shifted in this way can then be evaluated together across all targets and are plotted below in Supplementary Figure 5. The correlation between the score and ΔG_{bind}^0 in the entire PL-REX dataset has an R^2 of 0.46; this can be considered a good result for a scoring function that was not designed to produce scores that are comparable between different targets.

Supplementary Table 6*. Slope of the linear regression of the relationship between SQM 2.20 score and the experimental binding free energies ΔG_{bind}^0 (Score = $a \cdot \Delta G_{bind}^0 + b$).*

**Target**	**Slope *a***
01-CA2	3.13
02-HIV-PR	4.01
03-CK2	11.08
04-AR	5.38
05-Cath-D	4.03
06-BACE1	2.16
07-JAK1	2.76
08-Trypsin	5.74
09-CDK2	2.56
10-MMP12	5.61
**Average**	**4.64**

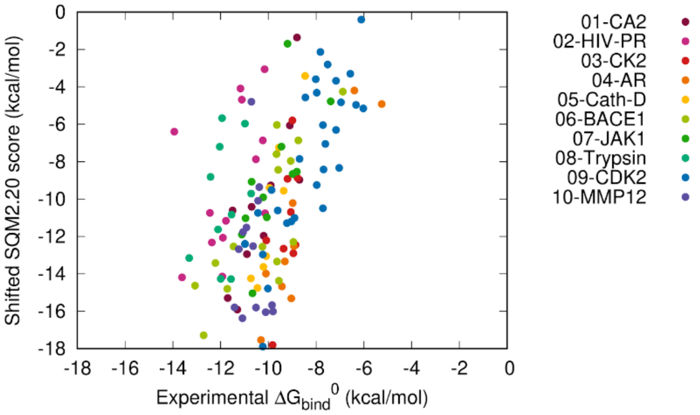

Supplementary Figure 5. *Plot of shifted SQM2.20 scores against the ΔG_{bind}^0. The same scale is used for the x and y axes to illustrate the slope of the dependence. Source data are provided as a Source Data files.*

S9) List of crystal structures used

Supplementary Table 7. *List of crystal structures of the P-L complexes used in this work. Along with the PDB code, we list the resolution (in Å) of the whole structure, and the real-space correlation coefficient (RSCC, ranging from 0 to 1) of the ligands indicating the reliability of the model with respect to the experimental electron density.*

PDB	Resolution	RSCC
	**01-CA2**	
5NXG	1.20	0.99
5NXI	1.16	0.95
5NXO	1.20	0.98
5NXP	1.25	0.99
5NXV	1.10	0.99
5NXW	1.10	0.97
5NY1	1.10	0.98
5NY3	1.40	0.97
5NY6	1.10	0.75
5NYA	1.20	0.99
	**02-HIV-PR**	
1HSG	2.00	n.d.
1HXW	1.80	n.d.
1IZH	1.90	n.d.
1T3R	1.20	n.d.
2Q54	1.85	0.91
2Q55	1.90	0.90
2Q5K	1.95	0.88
3EKX	1.97	0.75
3EL1	1.70	0.94
3NU3	1.02	0.97
3OXC	1.16	0.95
5HVP	2.00	n.d.
	**03-CK2**	
1F0Q	2.63	n.d.
1J91	2.22	n.d.
1M2P	2.00	n.d.
1M2Q	1.79	n.d.
1M2R	1.70	n.d.
1ZOE	1.77	n.d.
1ZOG	2.30	n.d.
1ZOH	1.81	n.d.
2OXD	2.30	0.95
2OXX	2.30	0.96
2OXY	1.81	0.96
3KXG	1.70	0.85
3KXH	1.70	0.80
3KXM	1.75	0.90
3KXN	2.00	0.88
3PVG	1.50	0.93

PDB	Resolution	RSCC
	**04-AR**	
1US0	0.66	1.00
2IKG	1.43	n.d.
2IKH	1.55	0.83
2IKI	1.47	0.95
2IKJ	1.55	0.97
4LAU	0.84	1.00
4LAZ	0.85	1.00
4LB3	0.80	1.00
4LB4	0.80	1.00
4LBR	0.80	1.00
4LBS	0.76	1.00
4QXI	0.87	0.98
4XZH	1.00	0.98
4XZI	2.45	0.91
	**05-Cath-D**	
6QBG	1.80	0.96
6QBH	1.85	0.96
6QCB	1.55	0.95
	**06-BACE1**	
5QCO	2.70	0.96
5QCP	2.45	0.94
5QCR	2.20	0.95
5QCT	2.05	0.96
5QCU	1.95	0.96
5QCV	2.25	0.94
5QCX	2.20	0.91
5QCY	2.15	0.91
5QCZ	2.30	0.92
5QD0	2.60	0.98
5QD1	2.40	0.95
5QD2	2.50	0.96
5QD3	2.46	0.94
5QD5	2.30	0.93
5QD9	2.60	0.97
5QDA	2.10	0.94

PDB	Resolution	RSCC
	**07-JAK1**	
4E4L	2.00	0.98
4E4N	1.90	0.97
4E5W	1.86	0.97
4EHZ	2.17	0.97
4EI4	2.22	0.96
4FK6	2.20	0.97
4I5C	2.10	0.97
4IVB	1.90	0.98
4IVC	2.35	0.97
4IVD	1.93	0.97
4K6Z	2.73	0.92
4K77	2.40	0.97
	**08-Trypsin**	
1K1I	2.20	0.96
1K1J	2.20	0.96
1K1L	2.50	0.94
1K1M	2.20	0.97
1K1N	2.00	0.96
2ZHD	1.94	0.84
2ZQ2	1.40	0.90
3LJJ	1.55	n.d.
3LJO	1.50	0.93
5MNG	0.86	0.99
5MO2	1.50	n.d.
6SY3	0.95	0.96
6T0M	1.51	0.93
6T0P	1.19	0.96
6T5W	1.13	0.96

PDB	Resolution	RSCC
**09-CDK2**		
3QQK	1.86	0.96
3QTQ	1.80	0.96
3QTR	1.85	0.95
3QTS	1.90	0.91
3QTU	1.82	0.97
3QTW	1.85	0.96
3QTX	1.95	0.95
3QTZ	2.00	0.98
3QU0	1.95	0.98
3QXP	1.75	0.97
3R8U	2.00	0.92
3R8V	1.90	0.94
3R8Z	1.85	0.91
3R9D	1.95	0.96
3R9N	1.75	0.95
3RAH	1.75	0.93
3RAK	1.75	0.98
3RAL	1.75	0.96
3RJC	1.85	0.92
3RK5	2.00	0.94
3RK7	1.80	0.88
3RK9	1.85	0.95
3RKB	2.00	0.95
3RMF	1.75	0.97
3RNI	1.95	0.91
3RPV	1.80	0.98
3RPY	1.90	0.97
3S00	1.80	0.91
3S0O	2.00	0.93
3S1H	1.75	0.97
3SQQ	1.85	0.97

PDB	Resolution	RSCC
**10-MMP12**		
3EHX	1.90	0.95
3EHY	1.90	0.98
3F15	1.70	0.97
3F16	1.16	0.94
3F17	1.10	0.96
3F18	1.13	0.94
3F19	1.13	0.98
3F1A	1.25	0.97
3LK8	1.80	0.98
3N2U	1.81	0.95
3N2V	1.55	0.96
3NX7	1.80	0.98
3RTS	1.81	0.97
3RTT	1.82	0.91
4GUY	2.00	0.95
5LAB	1.34	0.96
6RD0	1.90	0.75
6RLY	2.20	0.76

Total average	0.94
Total minimum	0.75

10) Plots of all scoring functions

Supplementary Note 9. *Plots of the score against experimental binding free energies for all the scoring functions discussed in the paper.*
This section contains plots of the score against the experimental binding free energy for each target, computed with the additional scoring functions on the PL-REX geometry, as Supplementary Figures 6 – 31. The overall comparison of these scoring functions is presented in Figure 1. of the paper and tables of the results are available here in Supplementary Tables 1 and 3A. The data used to generate these plots are available in the Supplementary Data file (directory raw_scores) as well as in the repository associated with the paper: (https://doi.org/10.5281/zenodo.8182922).

Supplementary Figure 6: Plots of the **SQM2.20** score against the experimental binding energy for each target.

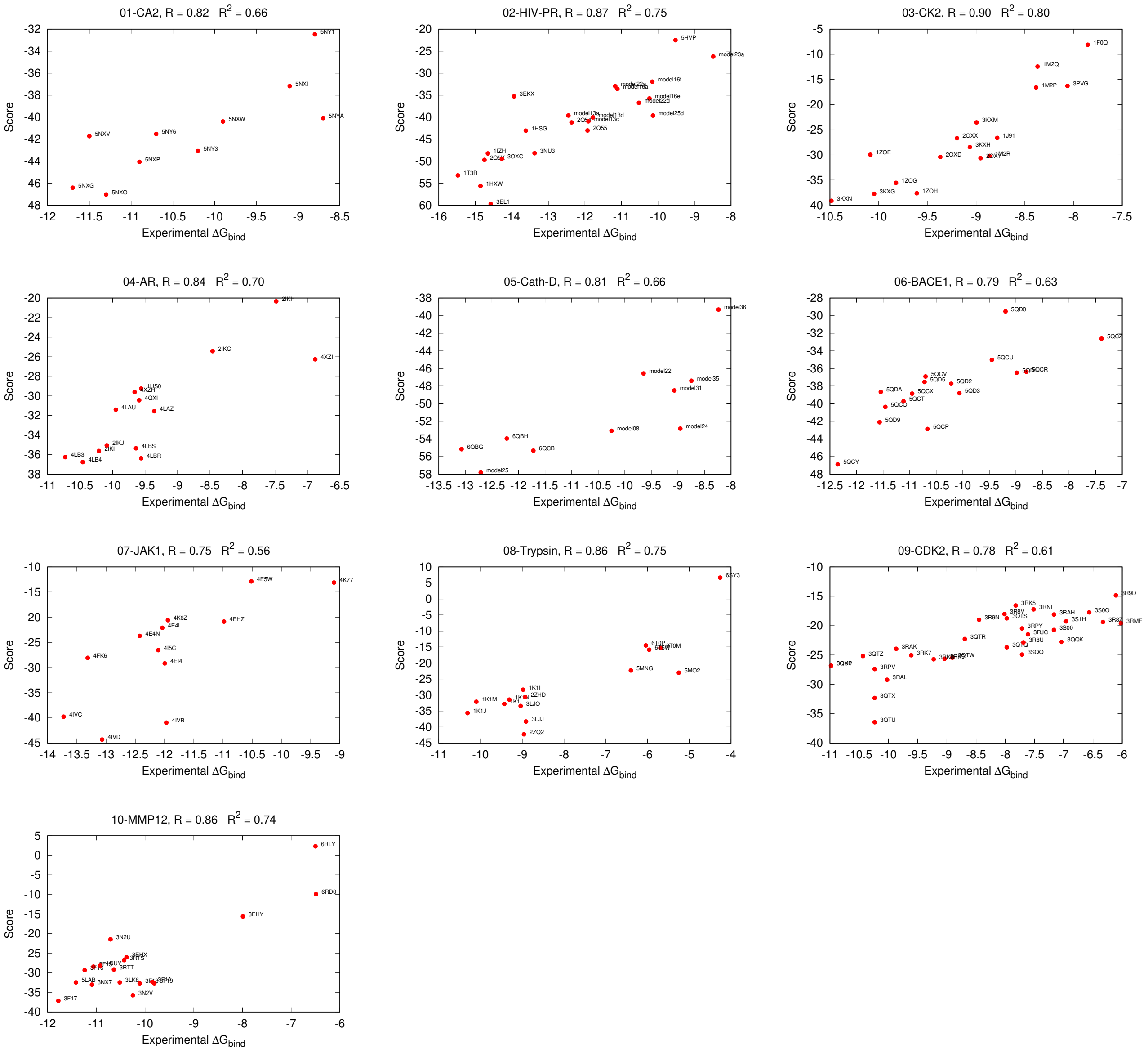

Supplementary Figure 7: Plots of the **AMBER/IGB7** score against the experimental binding energy for each target.

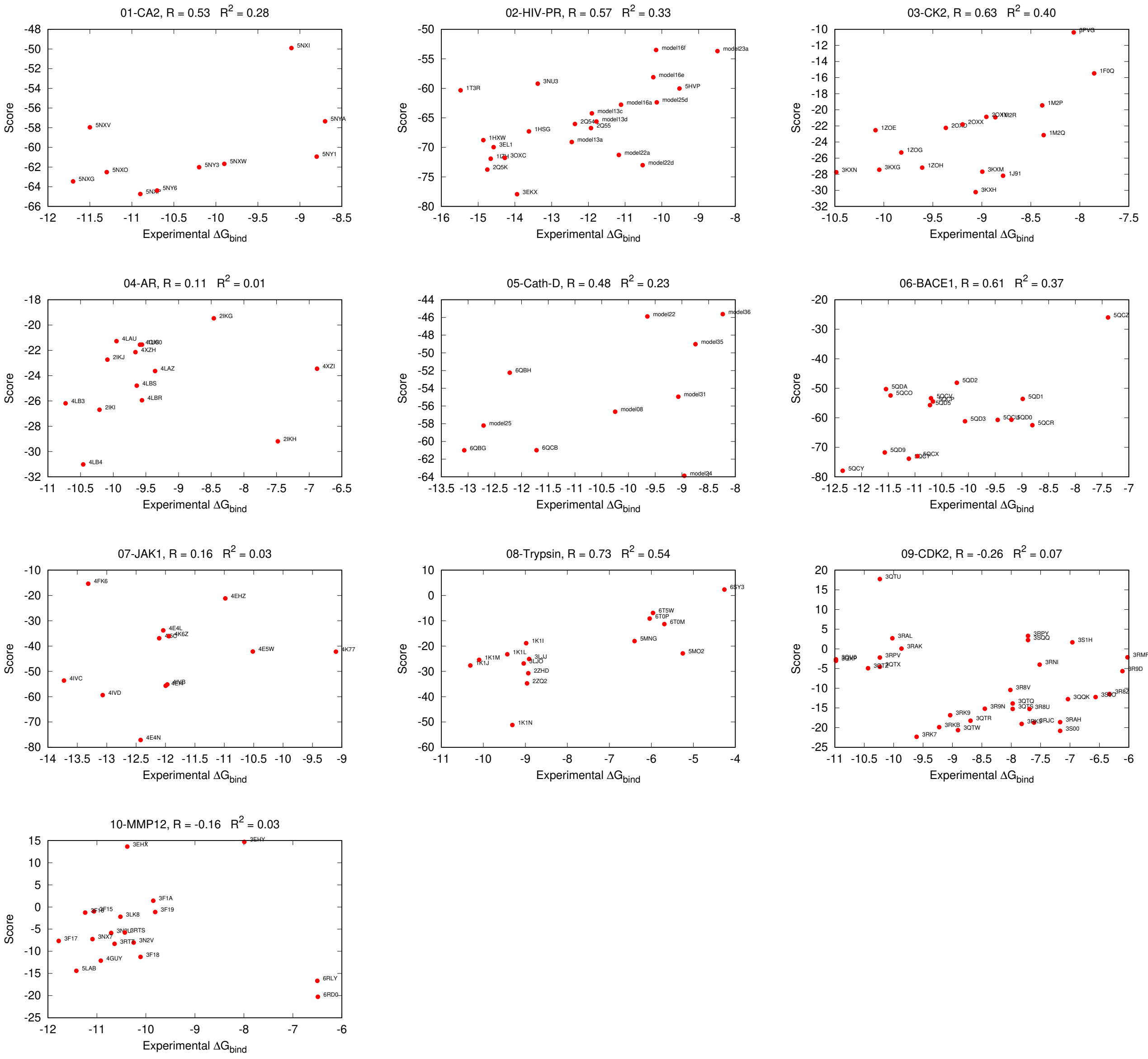

Supplementary Figure 8: Plots of the **GlideSP** score against the experimental binding energy for each target.

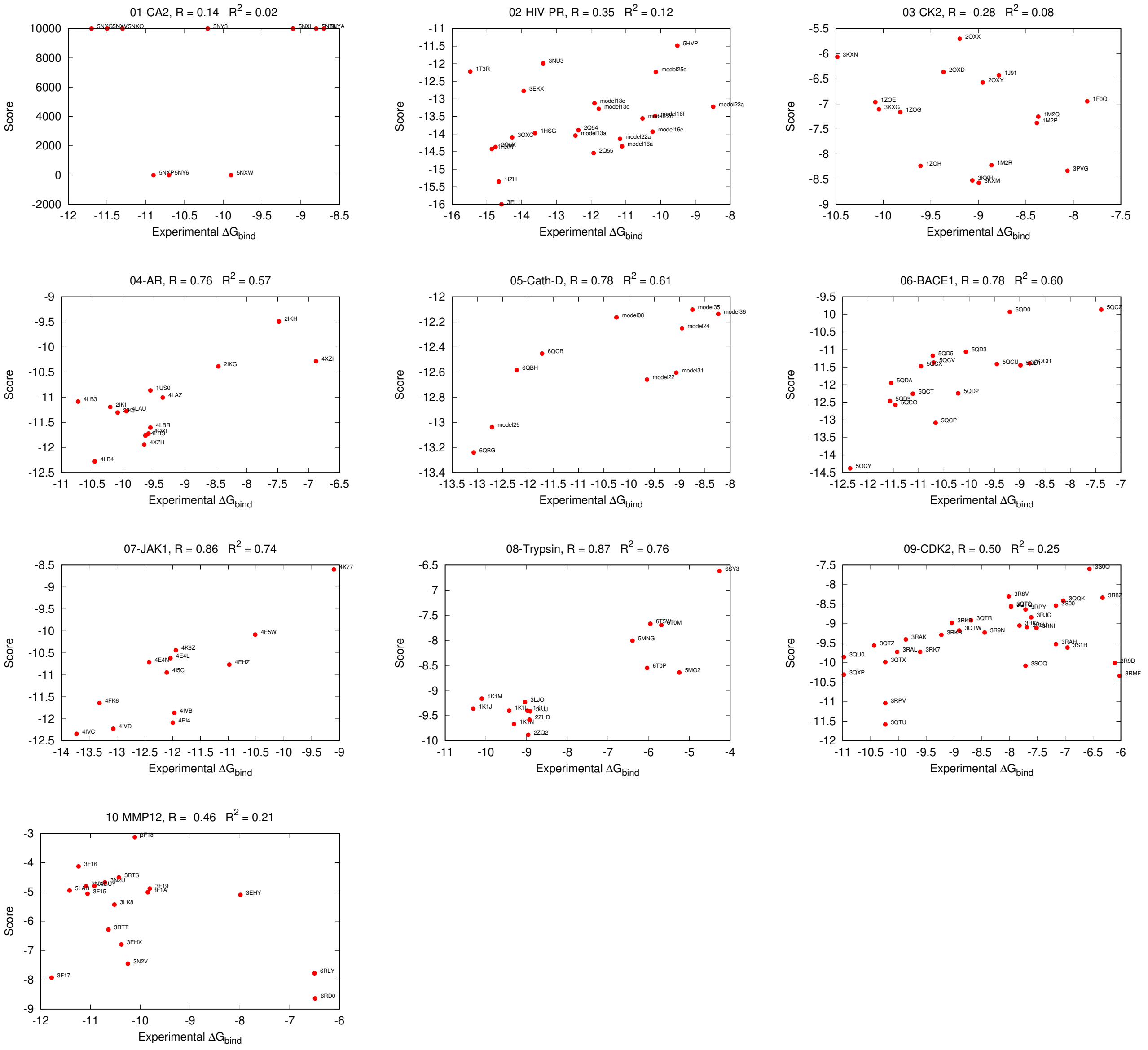

Supplementary Figure 9: Plots of the **GlideSP-min** score against the experimental binding energy for each target.

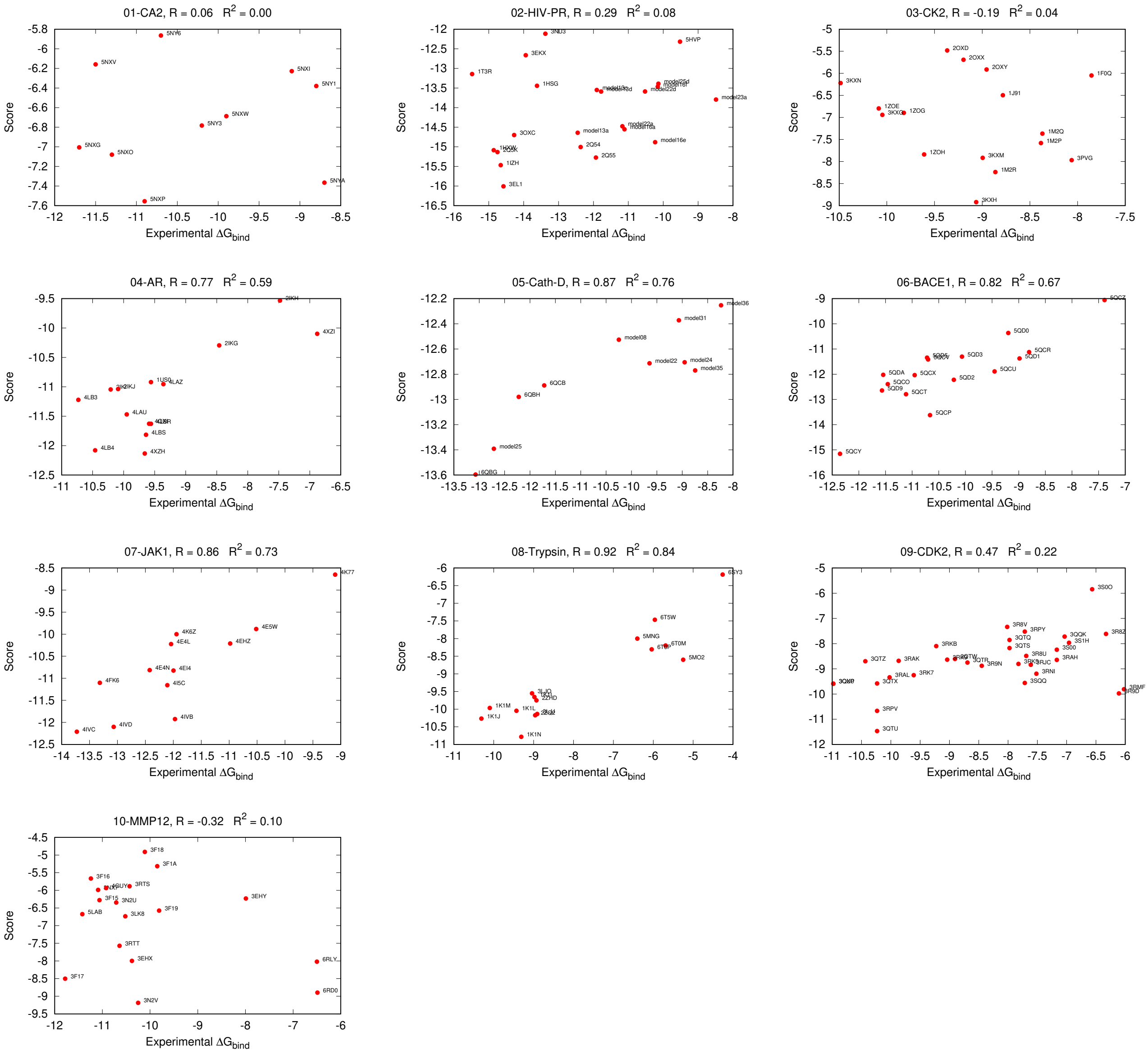

Supplementary Figure 10: Plots of the **GlideXP** score against the experimental binding energy for each target.

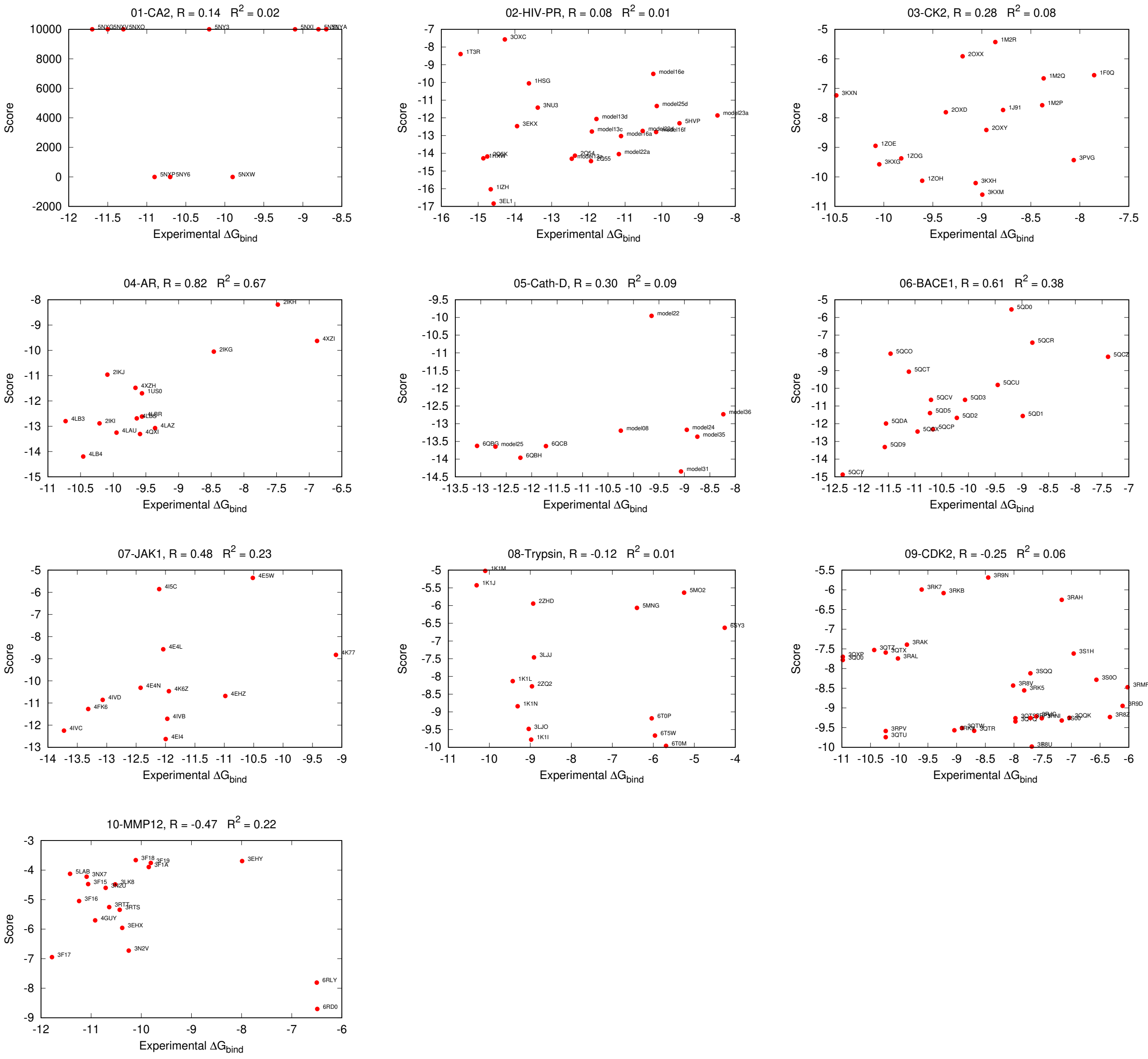

Supplementary Figure 11: Plots of the **GlideXP-min** score against the experimental binding energy for each target.

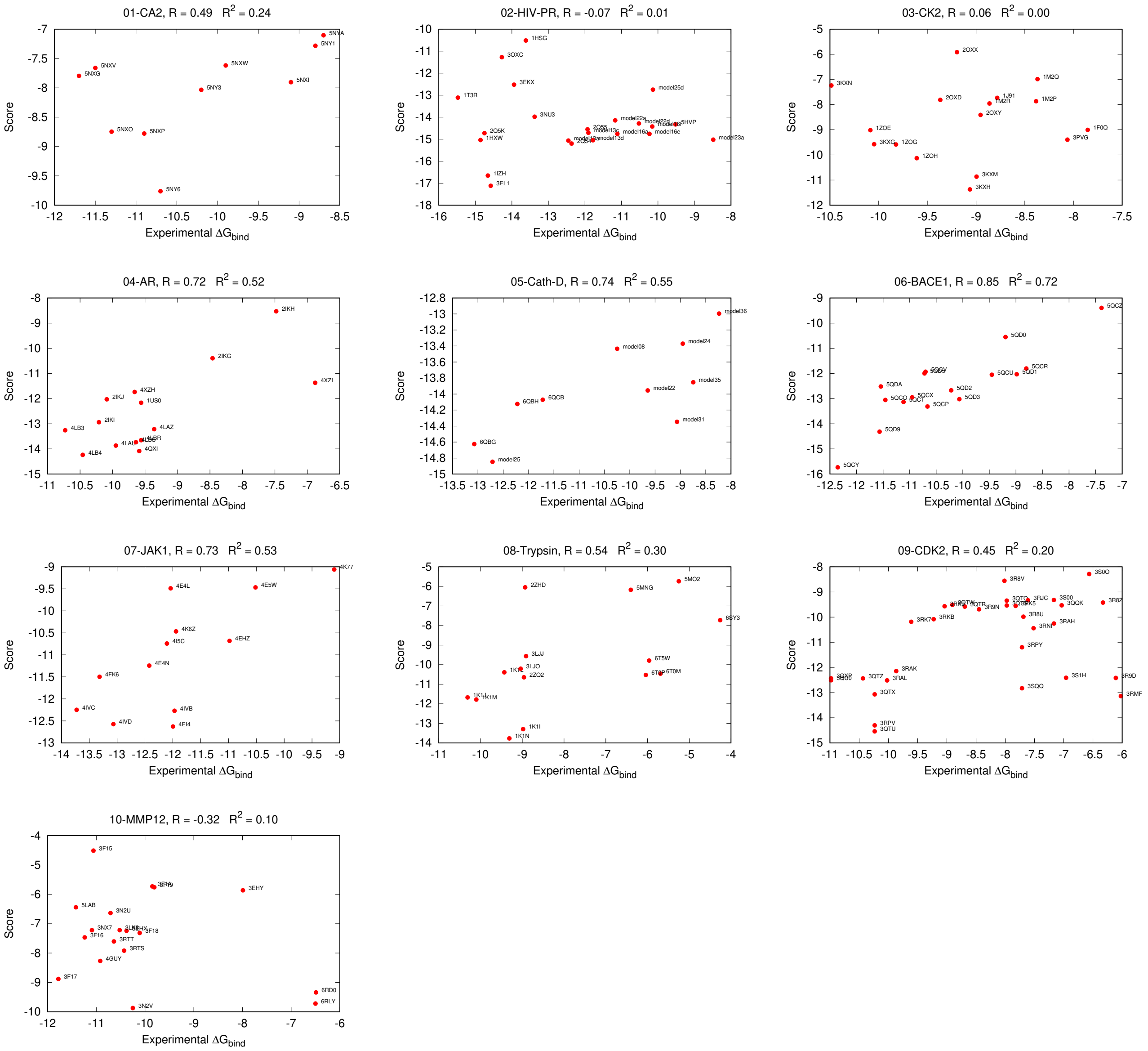

Supplementary Figure 12: Plots of the **Plants PLP** score against the experimental binding energy for each target.

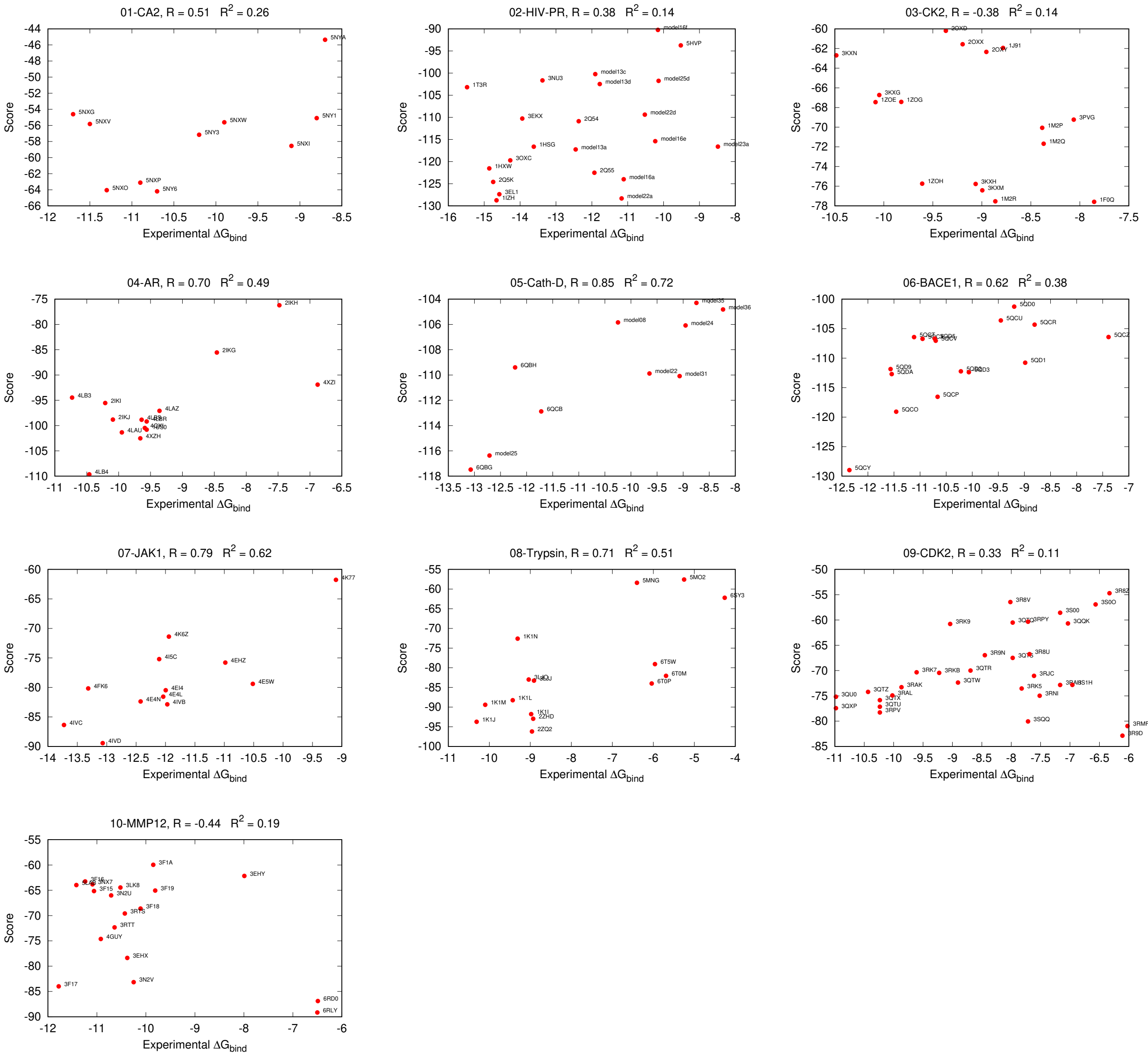

Supplementary Figure 13: Plots of the **Plants ChemPLP** score against the experimental binding energy for each target.

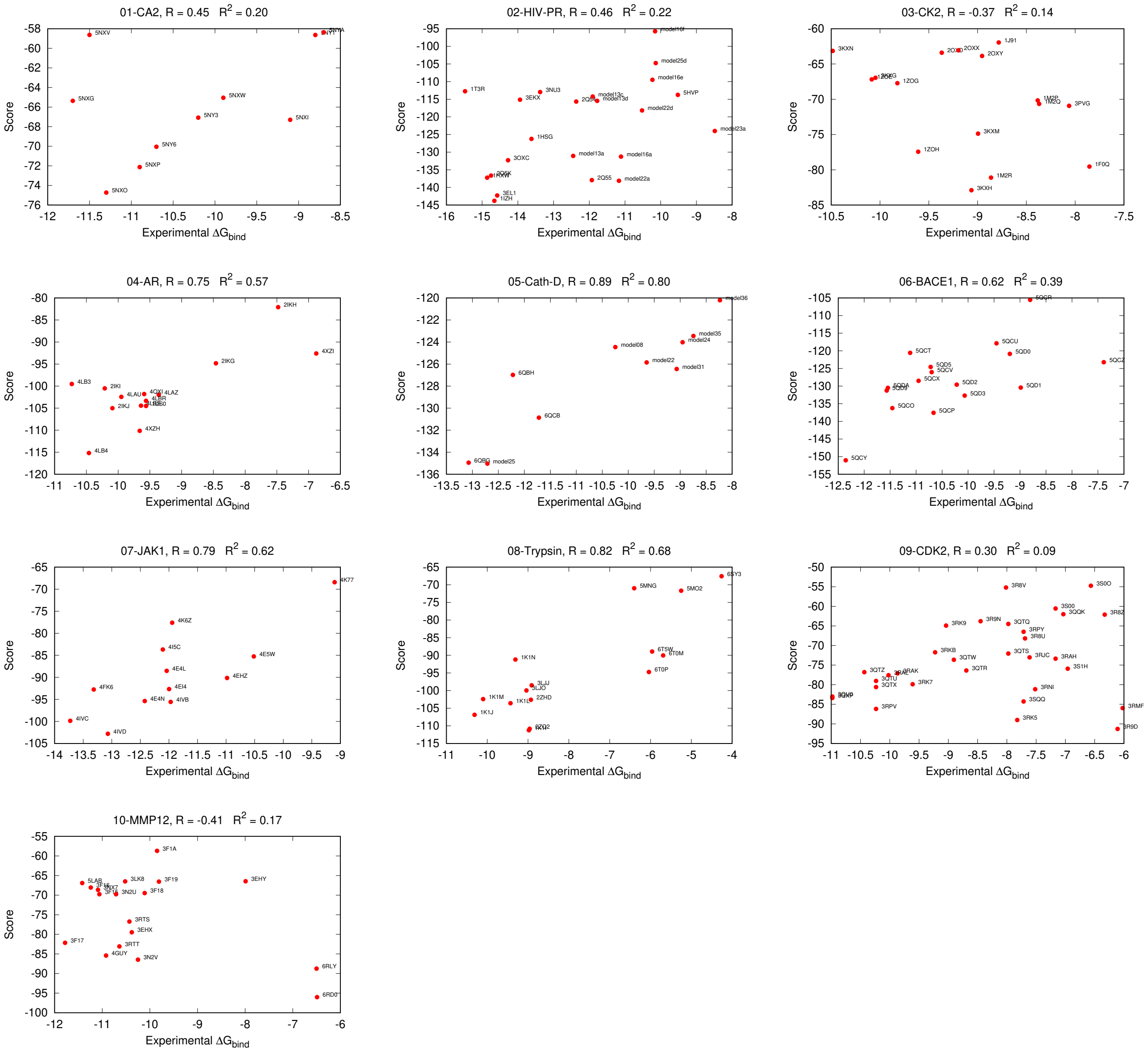

Supplementary Figure 14: Plots of the **X-Score** score against the experimental binding energy for each target.

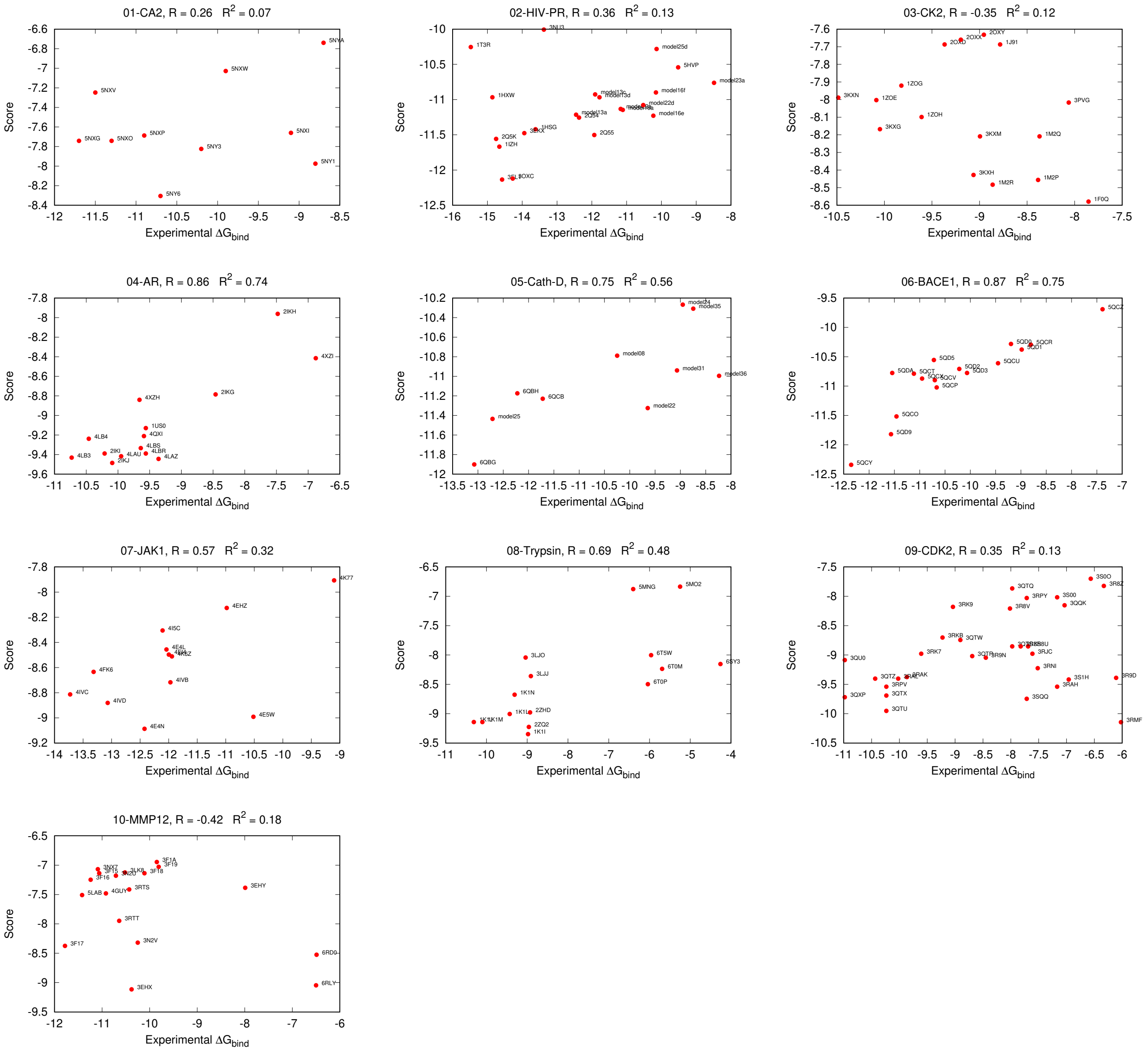

Supplementary Figure 15: Plots of the **X-Score HMS** score against the experimental binding energy for each target.

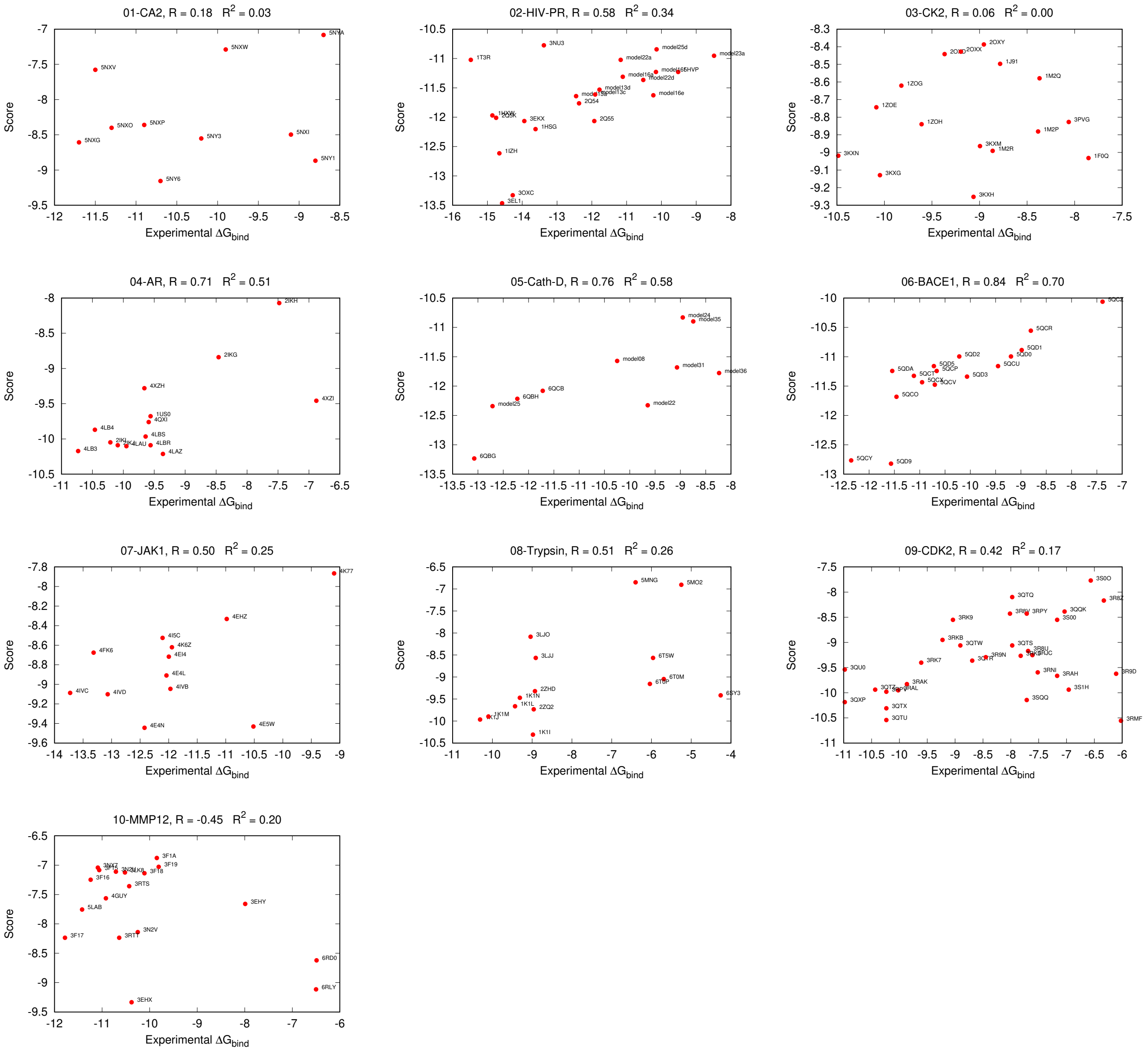

Supplementary Figure 16: Plots of the **X-Score HPS** score against the experimental binding energy for each target.

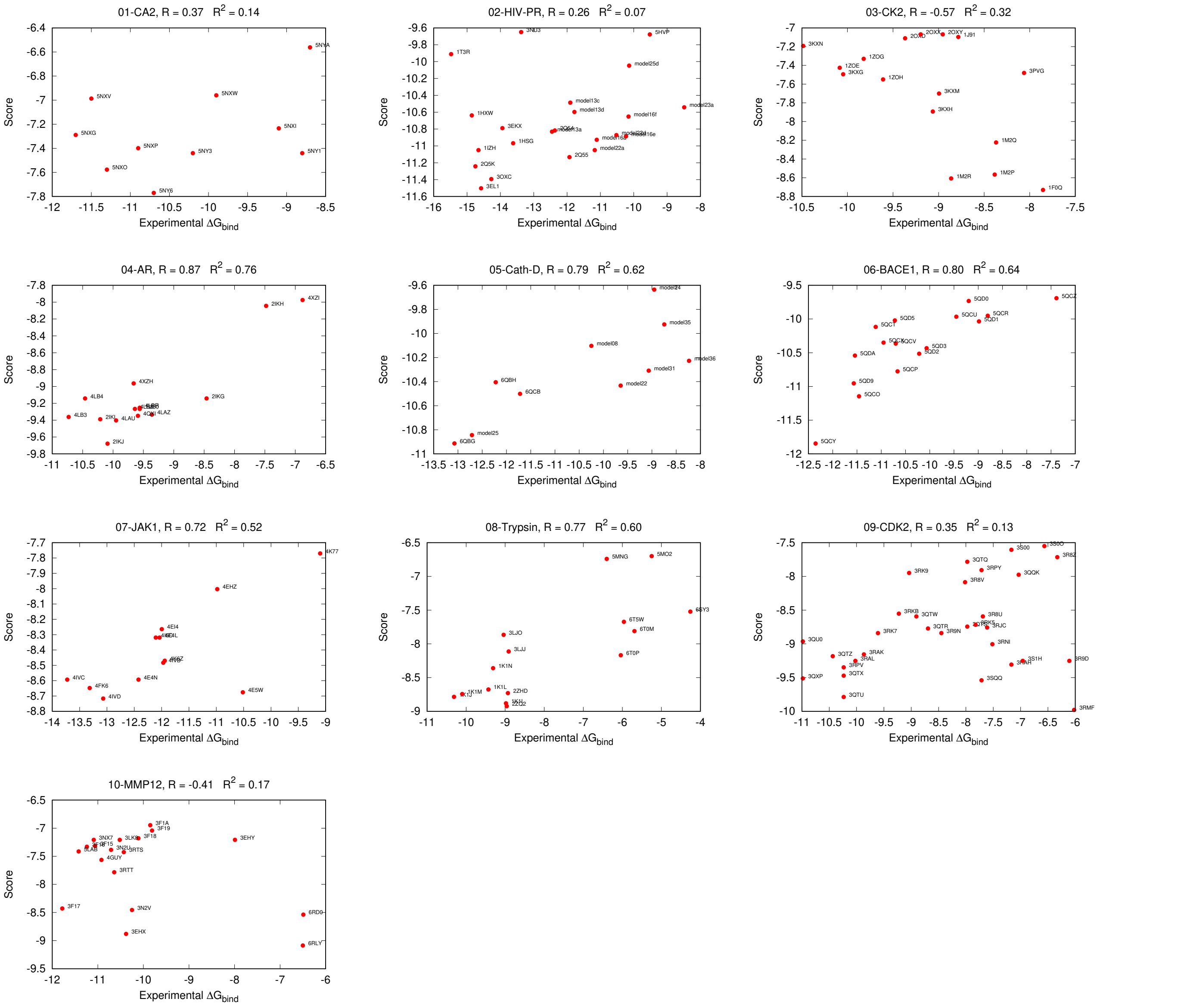

Supplementary Figure 17: Plots of the **X-Score HSS** score against the experimental binding energy for each target.

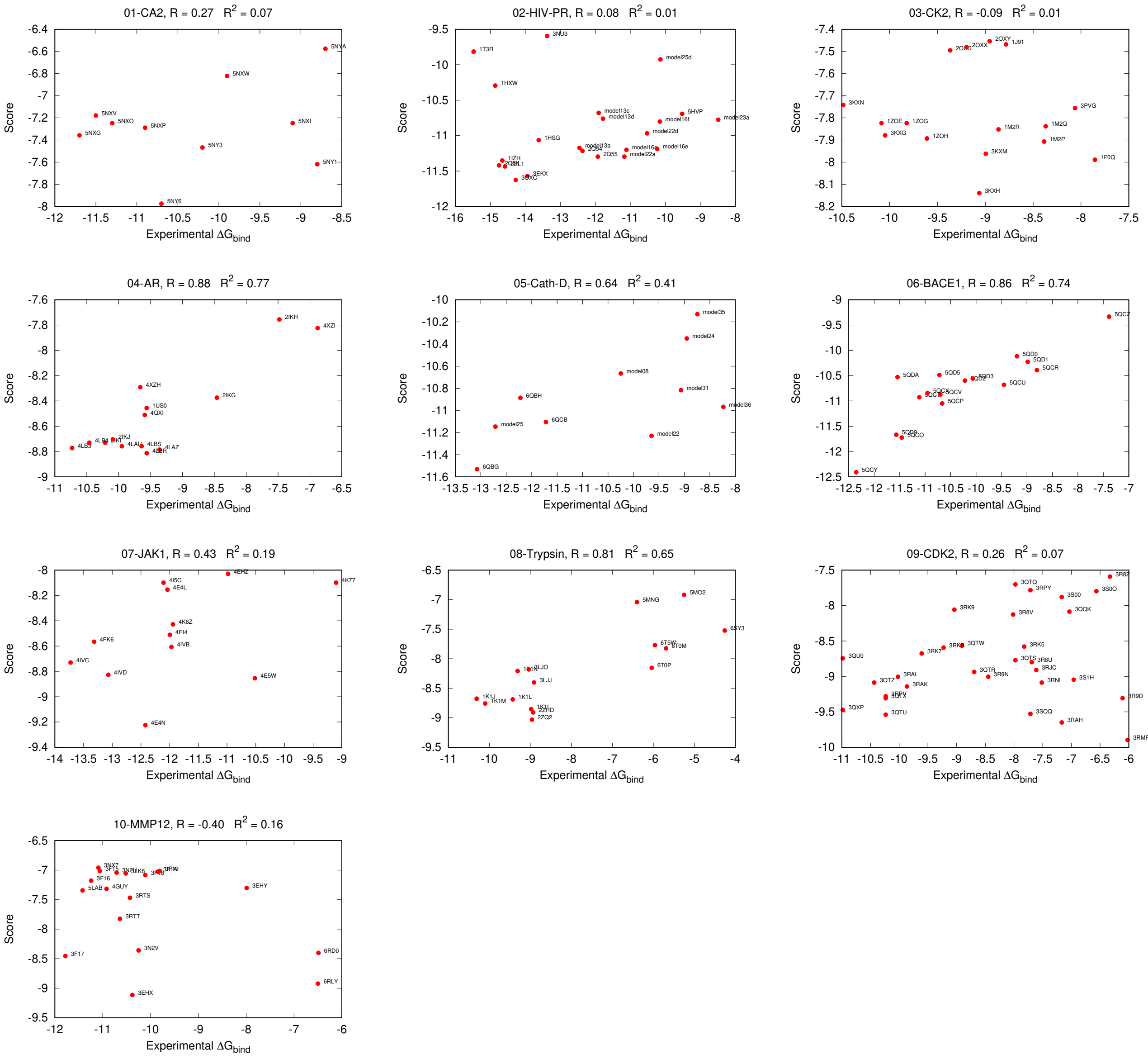

Supplementary Figure 18: Plots of the **Gold ChemPLP** score against the experimental binding energy for each target. GOLD scores have the opposite sign to the binding energy (the higher the score, the better the binding). To make the plots comparable to others, a negative of the score is plotted.

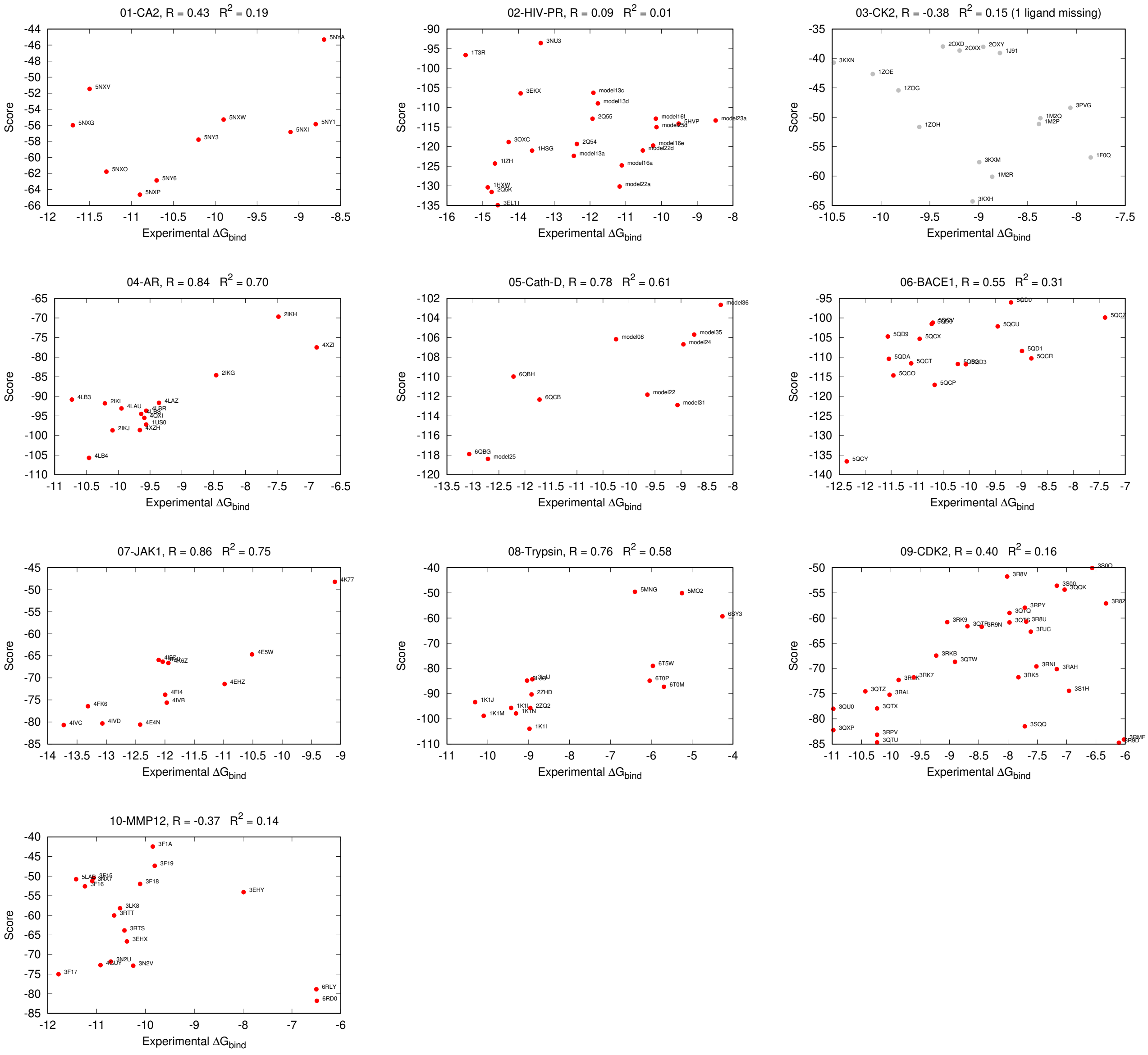

Supplementary Figure 19: Plots of the **Gold ASP** score against the experimental binding energy for each target. GOLD scores have the opposite sign to the binding energy (the higher the score, the better the binding). To make the plots comparable to others, a negative of the score is plotted.

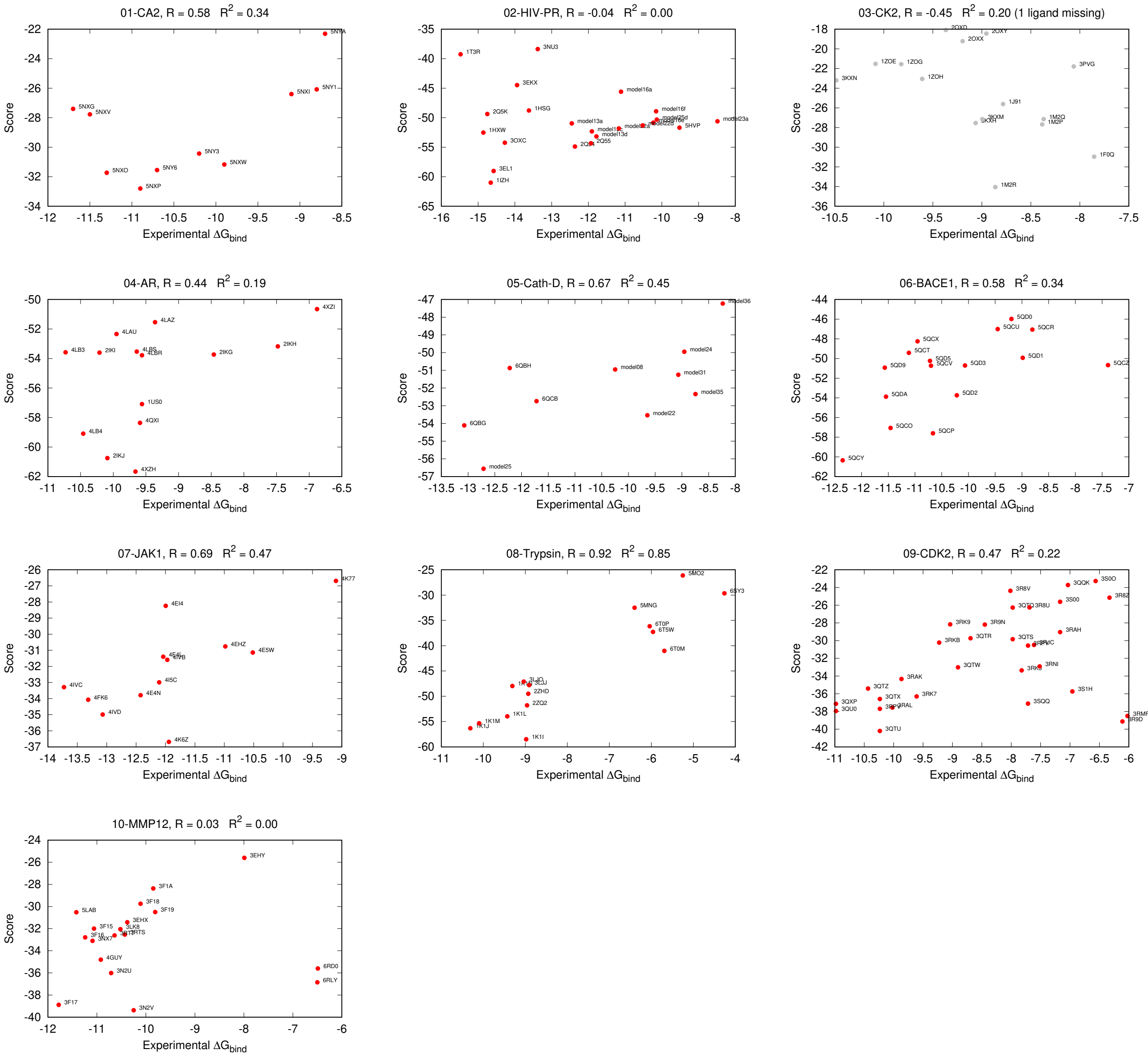

Supplementary Figure 20: Plots of the **Gold GS** score against the experimental binding energy for each target. GOLD scores have the opposite sign to the binding energy (the higher the score, the better the binding). To make the plots comparable to others, a negative of the score is plotted.

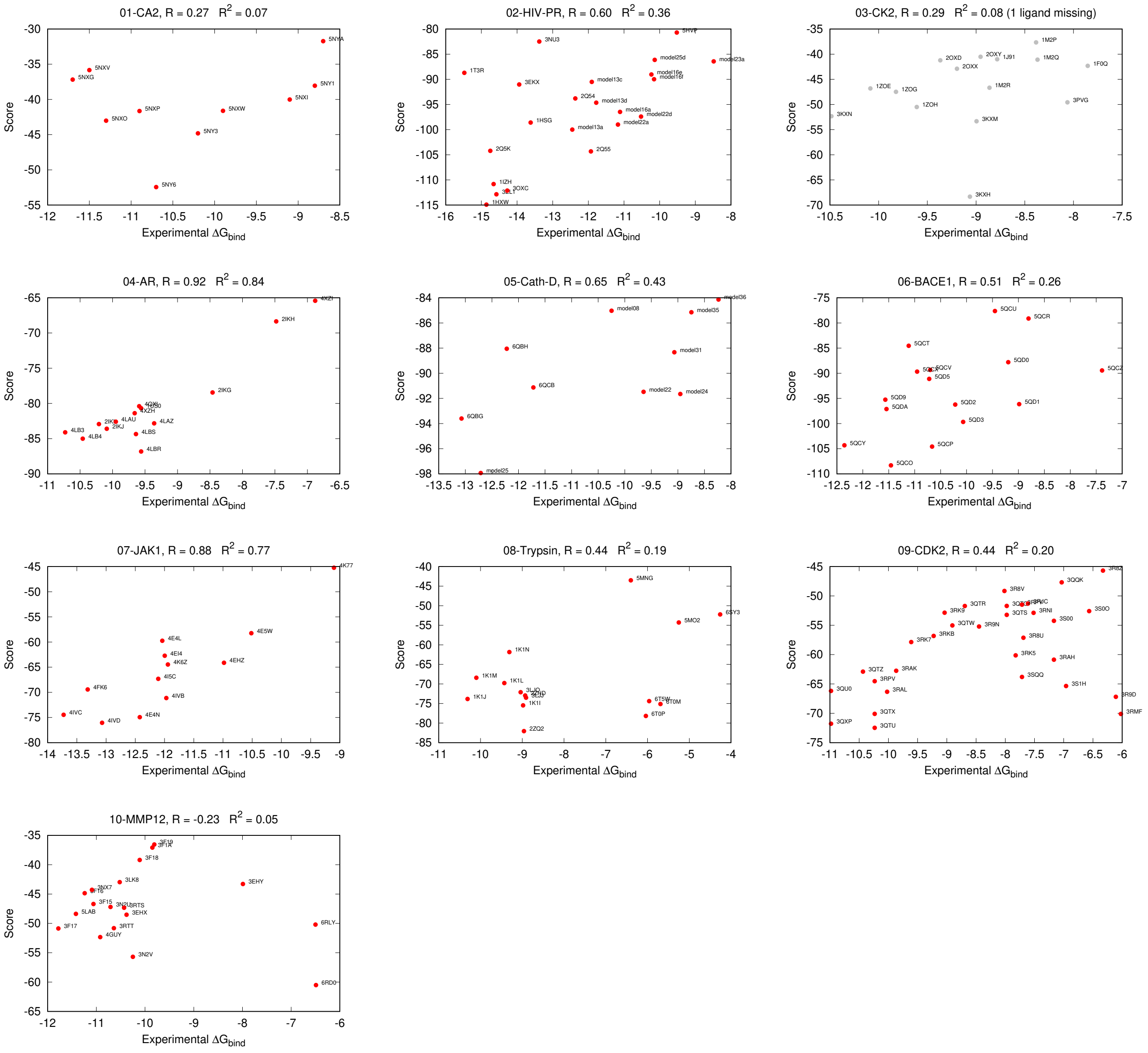

Supplementary Figure 21: Plots of the **Gold CHS** score against the experimental binding energy for each target. GOLD scores have the opposite sign to the binding energy (the higher the score, the better the binding). To make the plots comparable to others, a negative of the score is plotted.

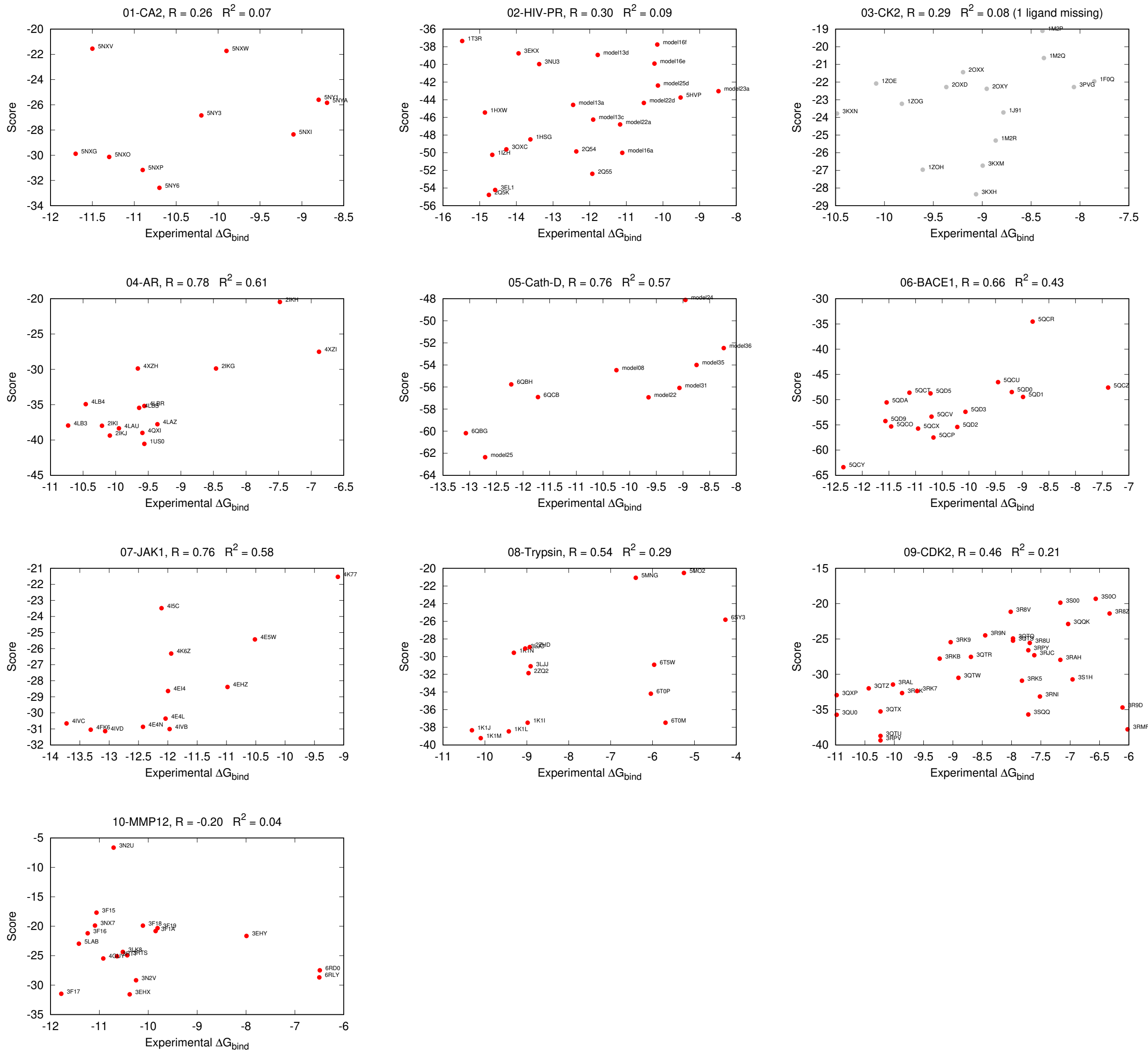

Supplementary Figure 22: Plots of the **Vina** score against the experimental binding energy for each target.

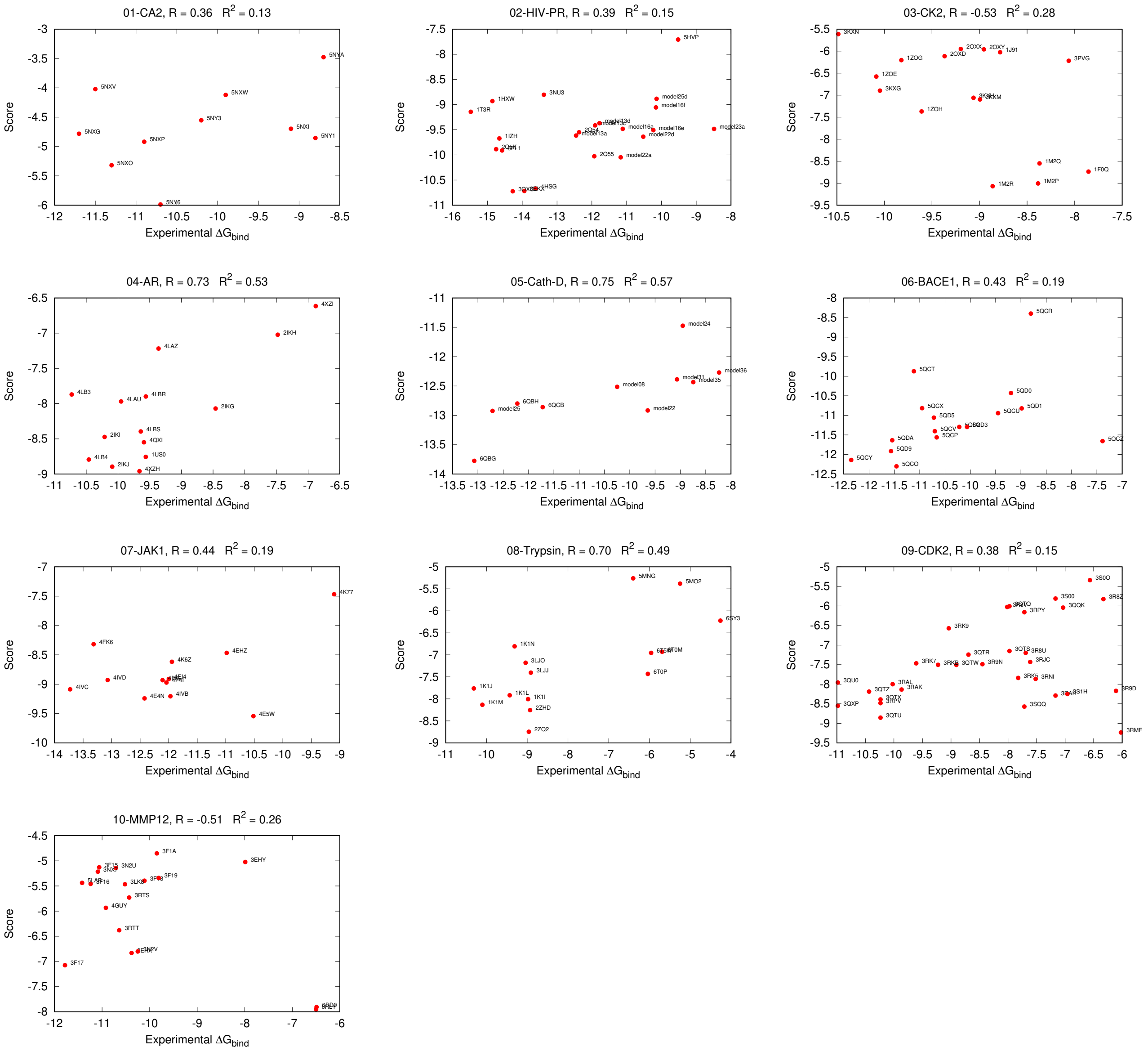

Supplementary Figure 23: Plots of the **Vinardo** score against the experimental binding energy for each target.

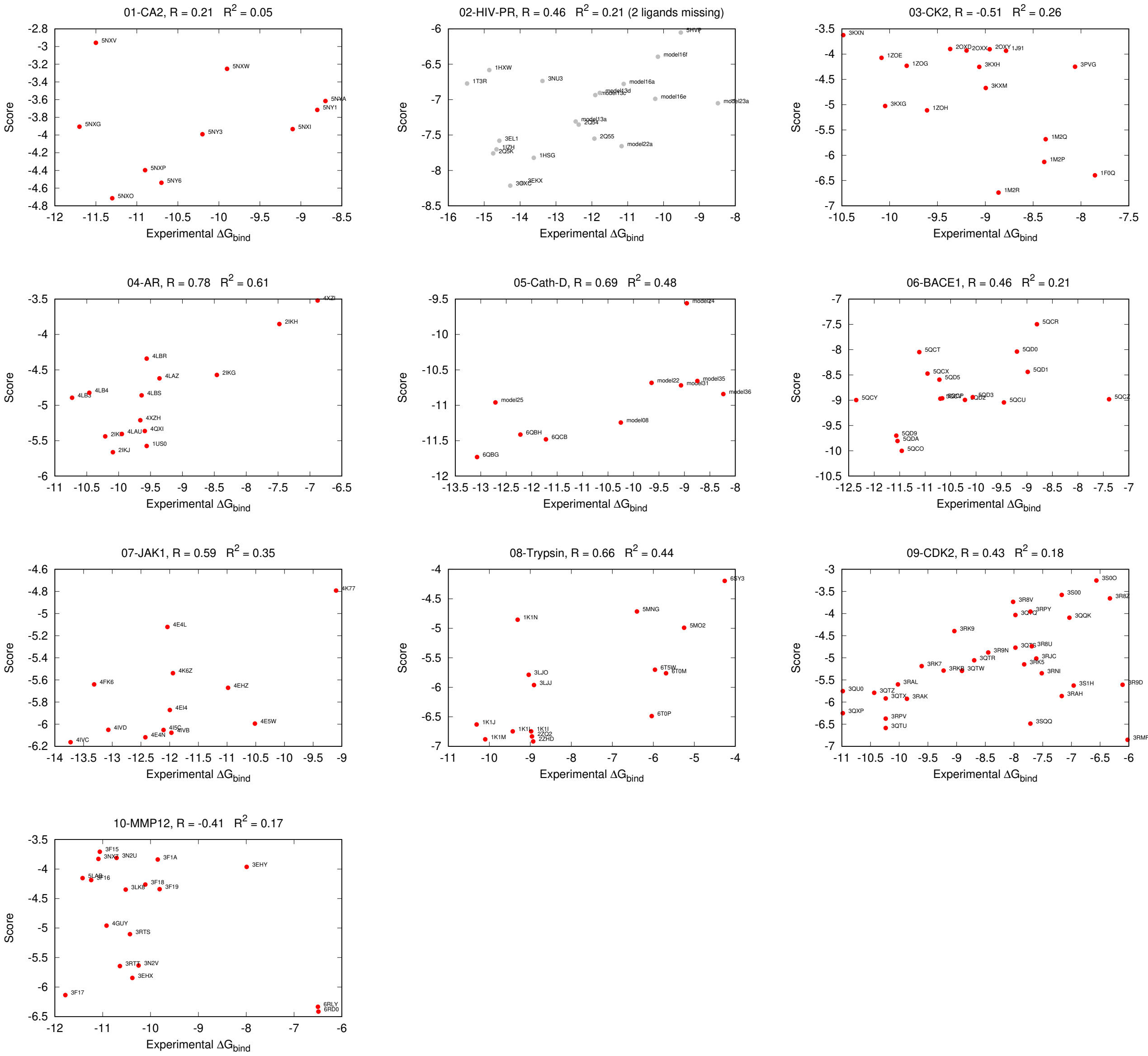

Supplementary Figure 24: Plots of the **Smina** score against the experimental binding energy for each target.

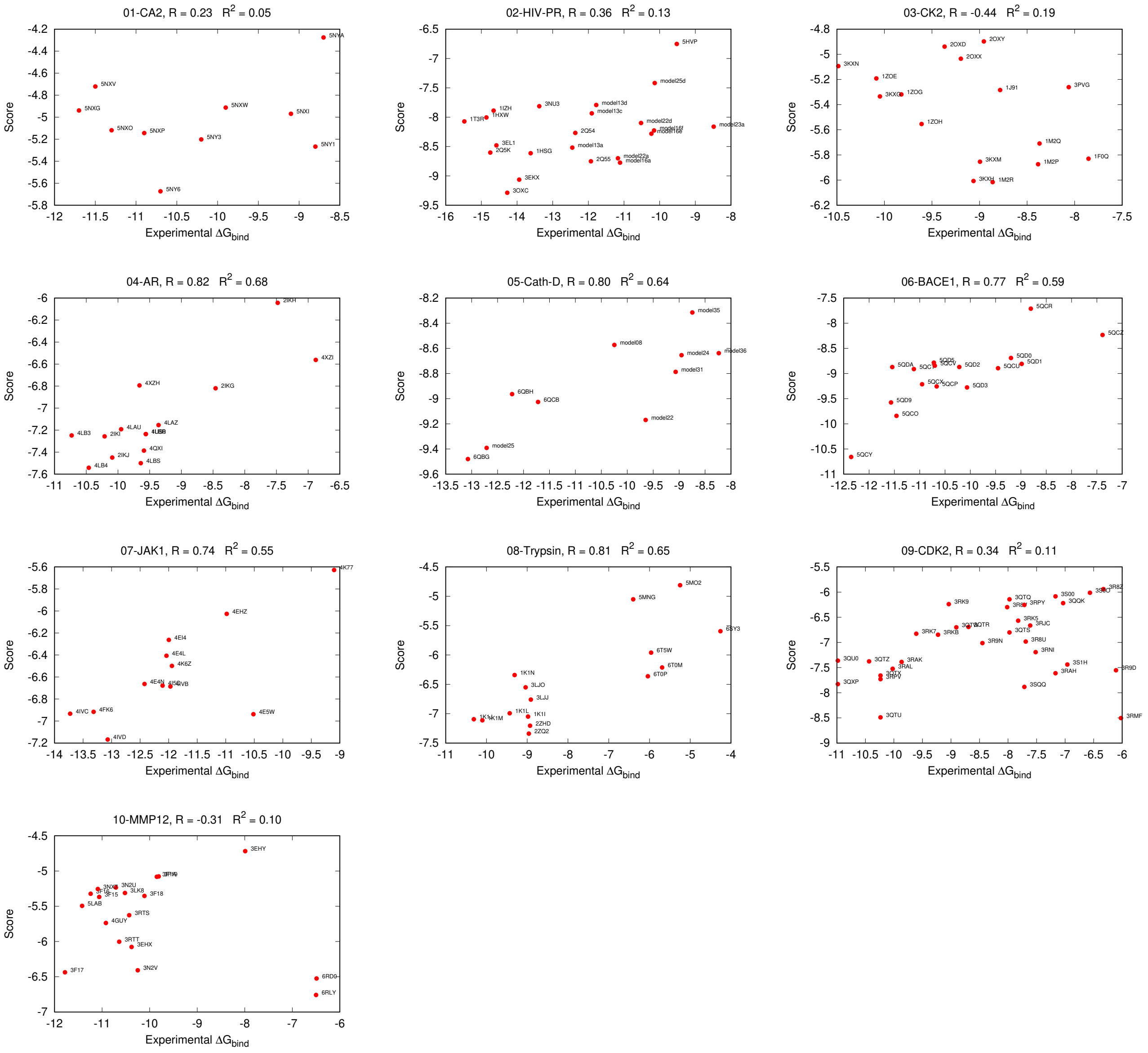

Supplementary Figure 25: Plots of the **Autodock4** score against the experimental binding energy for each target.

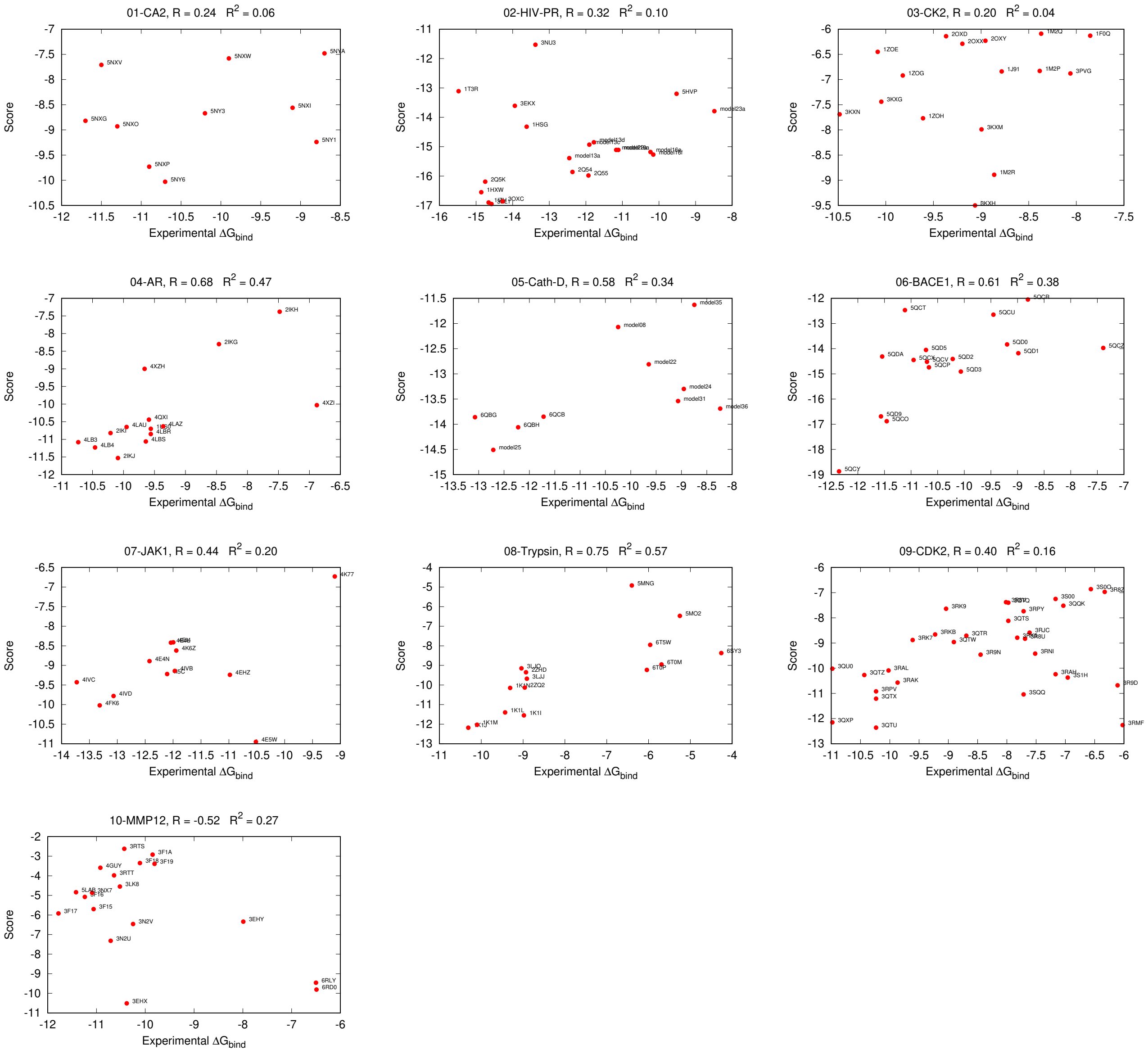

Supplementary Figure 26: Plots of the $\Delta_{\mathbf{Vina}}\mathbf{RF}_{20}$ score against the experimental binding energy for each target.

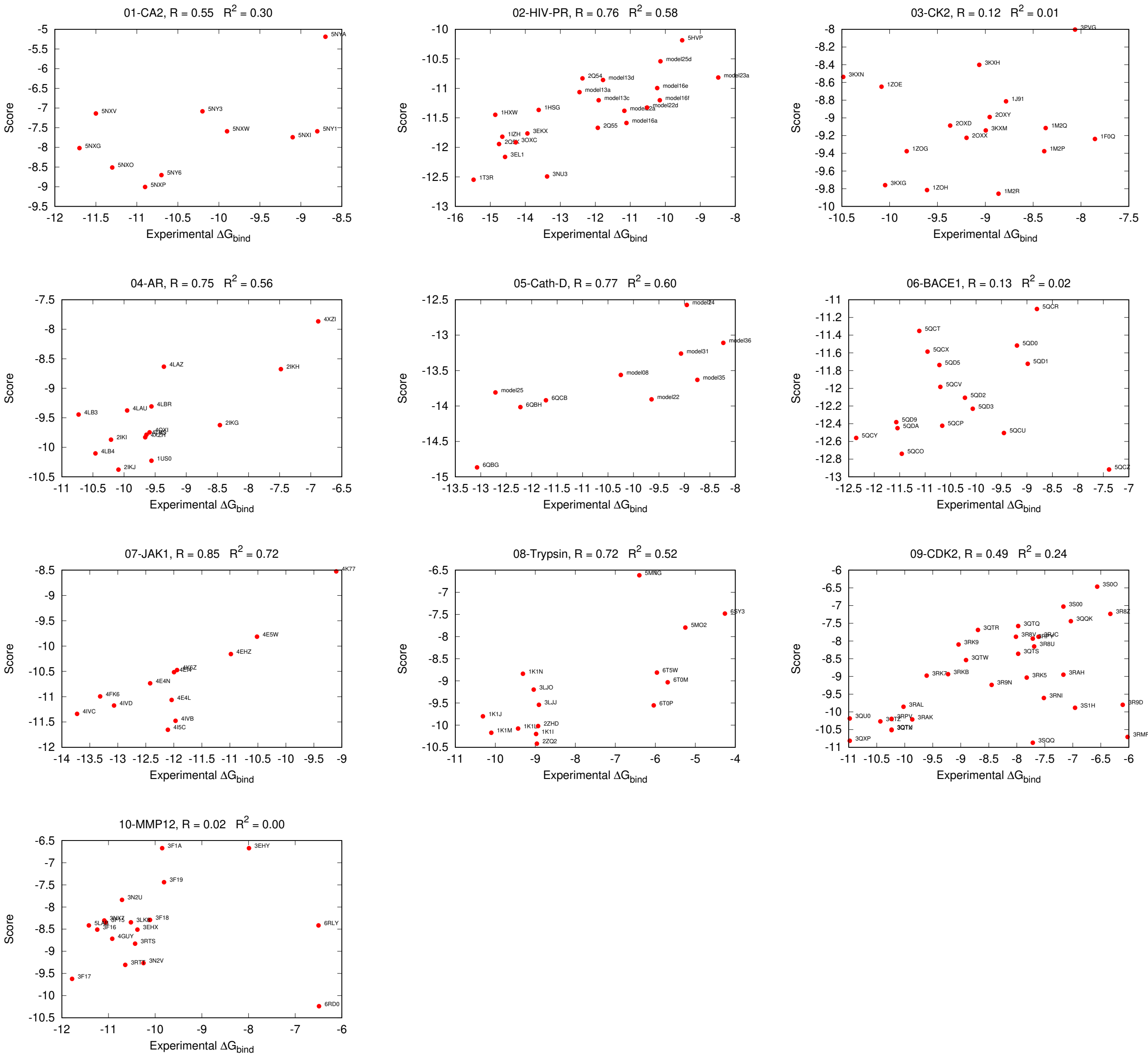

Supplementary Figure 27: Plots of the **NNScore2** score against the experimental binding energy for each target.

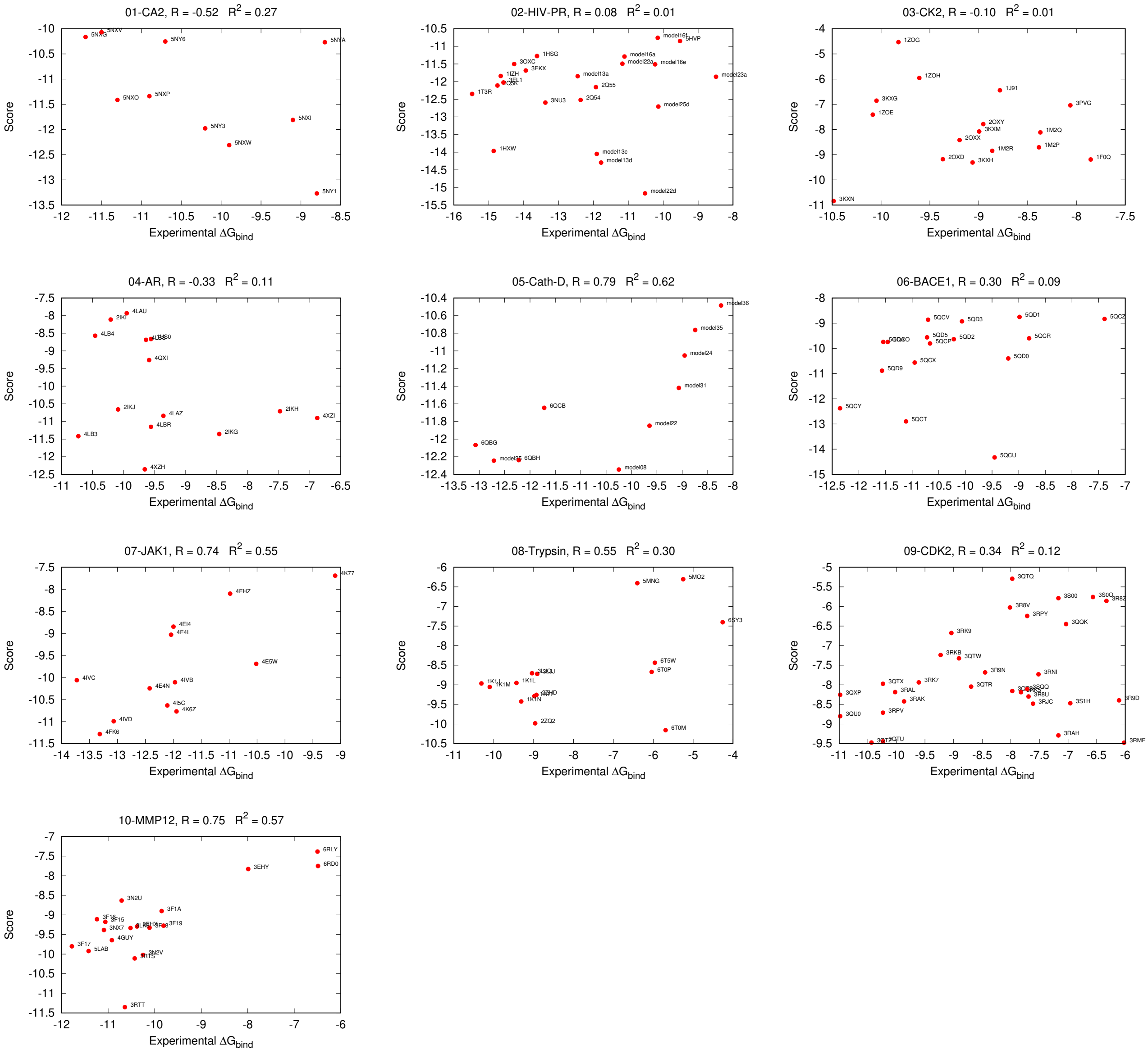

Supplementary Figure 28: Plots of the **RF-Score-VS** score against the experimental binding energy for each target.

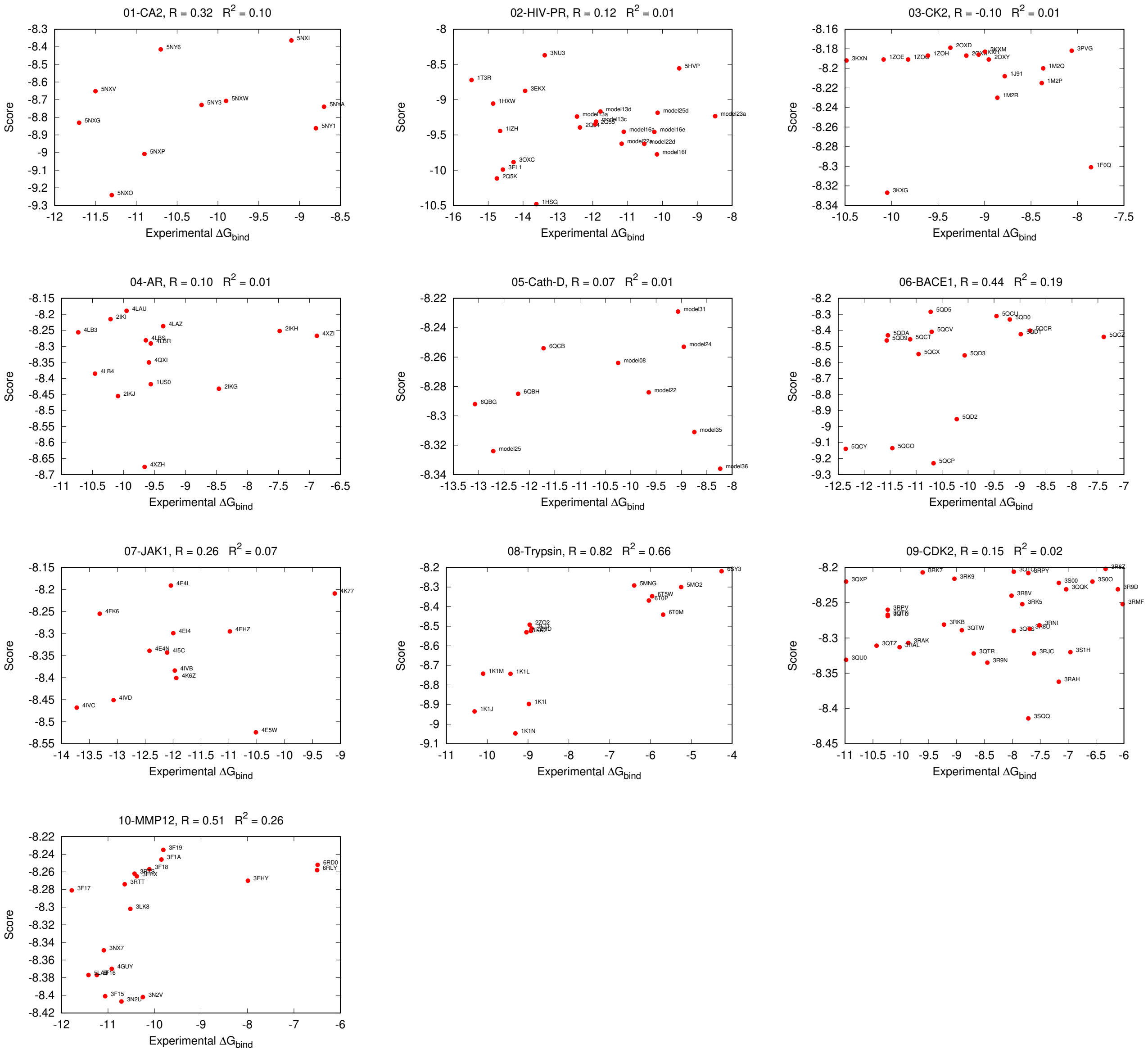

Supplementary Figure 29: Plots of the **Pafnucy** score against the experimental binding energy for each target.

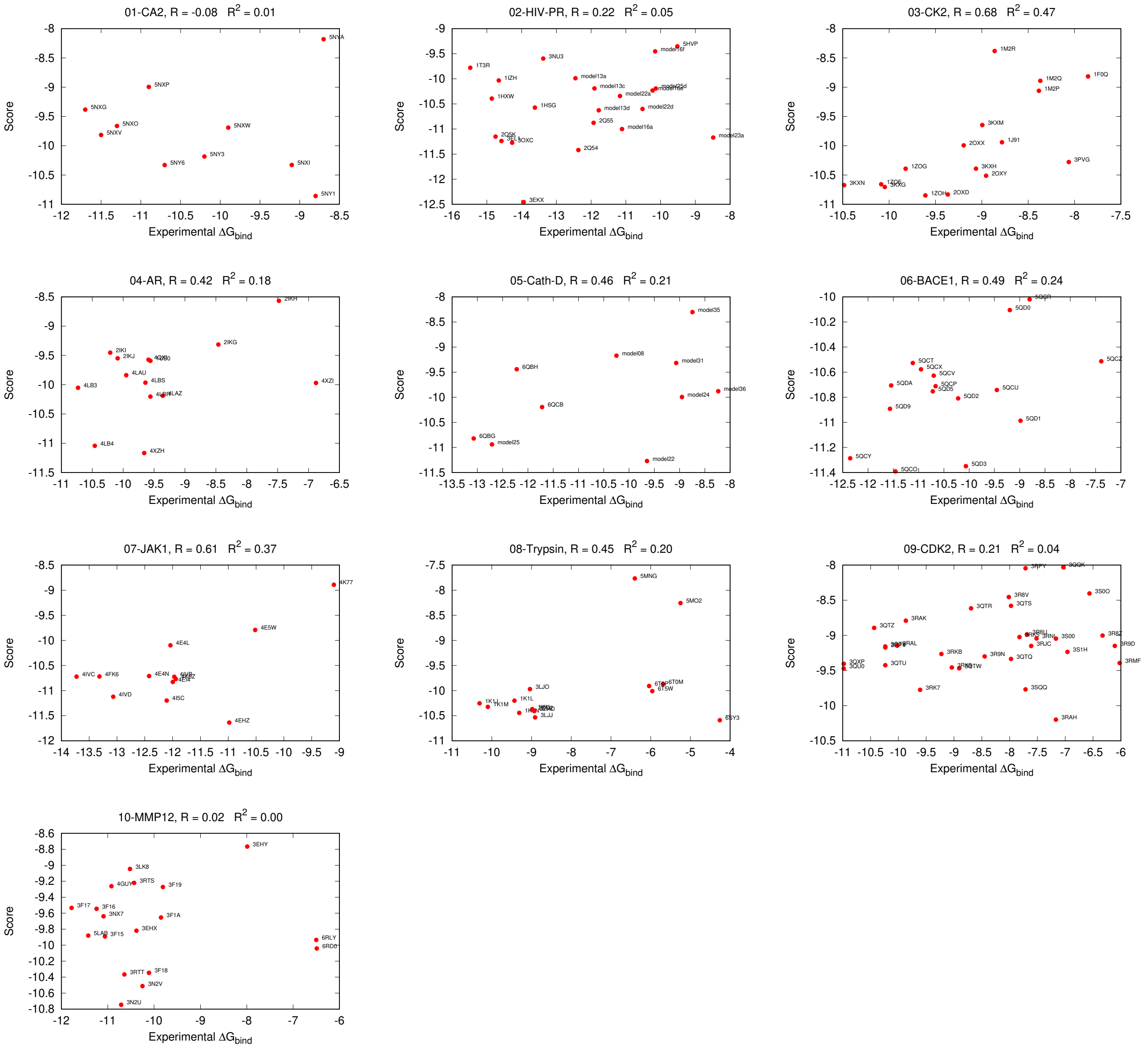

Supplementary Figure 30: Plots of the **SQM(default PM6-D3H4X/COSMO)** score against the experimental binding energy for each target.

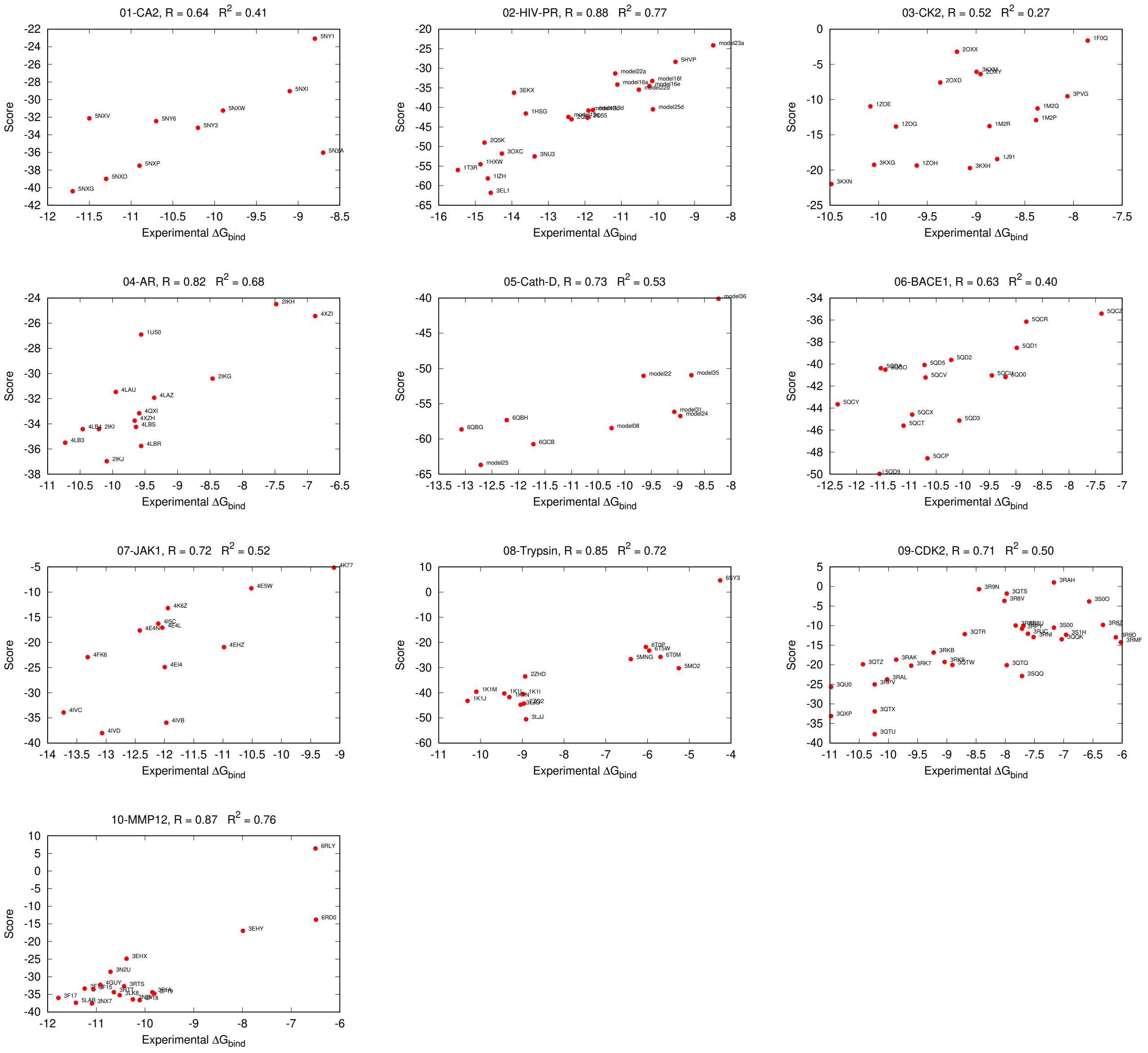

Supplementary Figure 31: Plots of the **SQM(PM7/COSMO)** score against the experimental binding energy for each target.

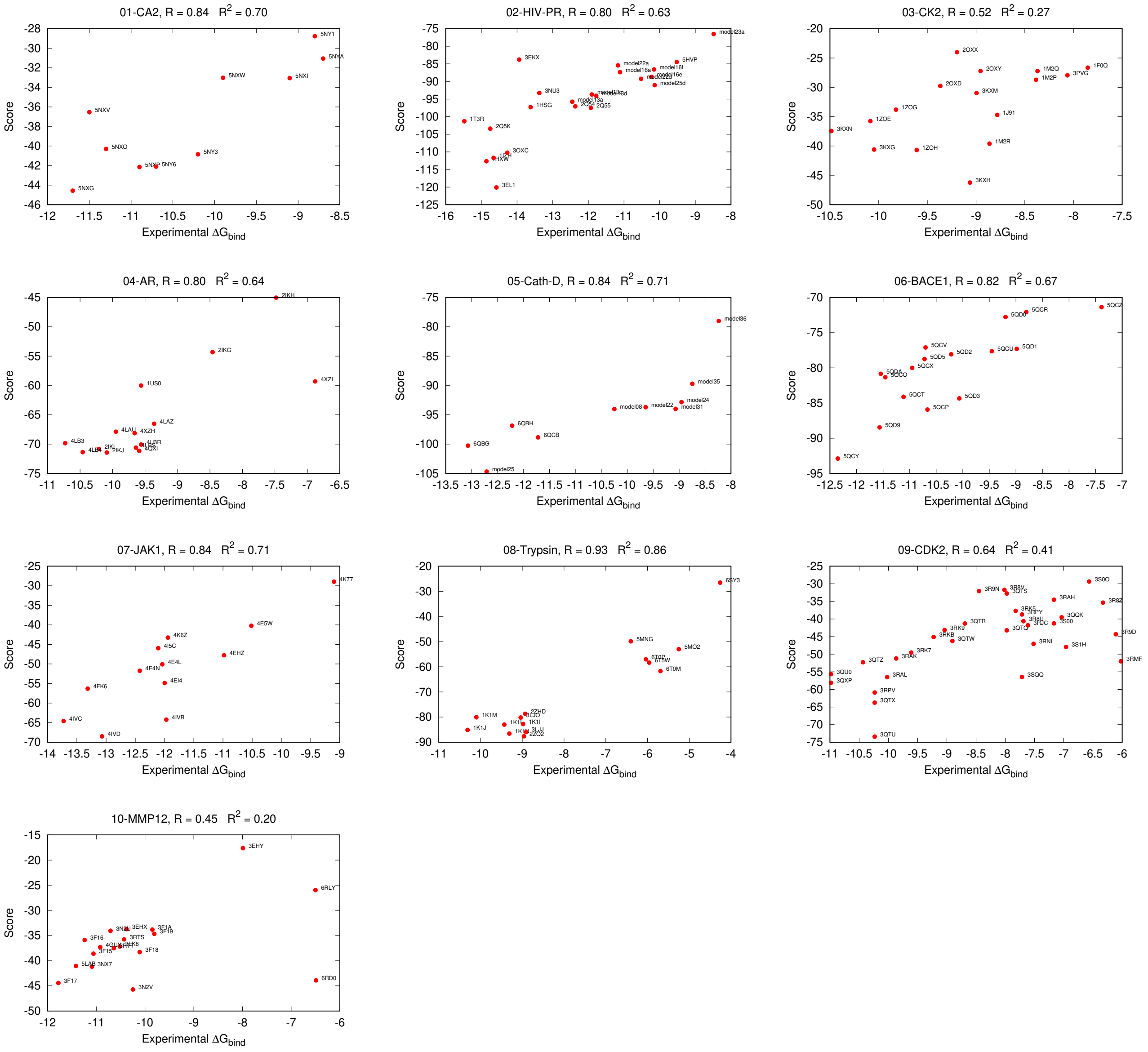

11) 2D structures of the ligands

Supplementary Note 10. *2D structures of the ligands and schematic drawings of the binding mode of the reference ligands.*

This section lists the 2D structures of the ligands. For each target, a schematic view of the binding pose in the protein-ligand complex selected as the representative structure used for scoring is also provided. The figures have been generated from the 3D structures available in the repository associated with the paper:
(https://doi.org/10.5281/zenodo.8182922).

Supplementary Figure 32: Target 01-CA2, schematic drawing of the binding pose in the crystal selected for scoring, PDB code 5NXG.

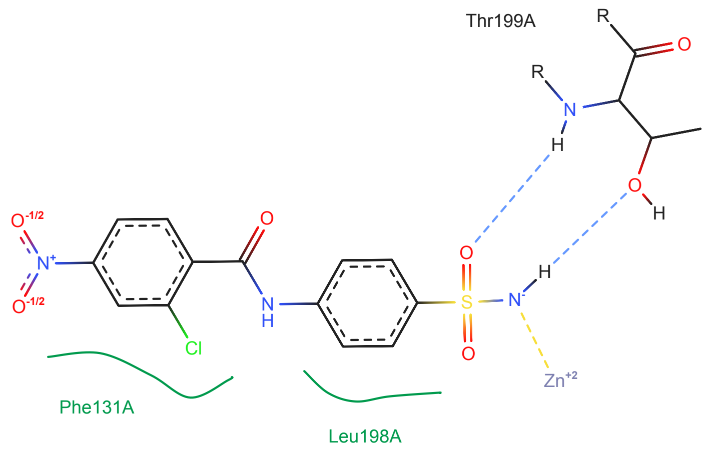

Supplementary Figure 33: Target 01-CA2, 2D structures of the ligands in the series and their binding free energies derived from the experiment.

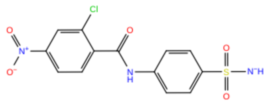

5NXG

ΔG_{bind} = -11.7 kcal/mol

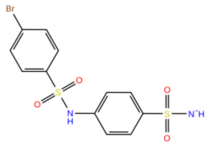

5NXI

ΔG_{bind} = -9.1 kcal/mol

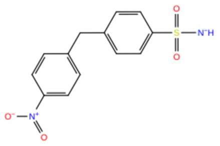

5NXO

ΔG_{bind} = -11.3 kcal/mol

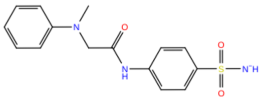

5NXP

ΔG_{bind} = -10.9 kcal/mol

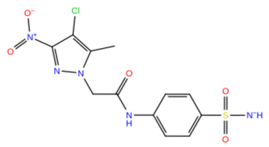

5NXV

ΔG_{bind} = -11.5 kcal/mol

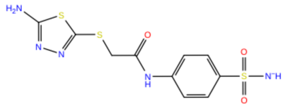

5NXW

ΔG_{bind} = -9.9 kcal/mol

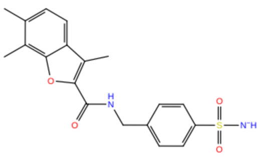

5NY1

ΔG_{bind} = -8.8 kcal/mol

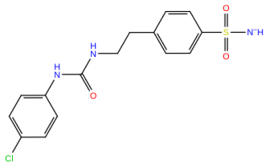

5NY3

ΔG_{bind} = -10.2 kcal/mol

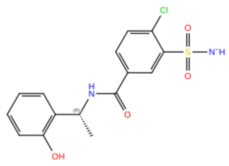

5NY6

ΔG_{bind} = -10.7 kcal/mol

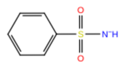

5NYA

ΔG_{bind} = -8.7 kcal/mol

Supplementary Figure 34: Target 02-HIV-PR, schematic drawing of the binding pose in the crystal selected for scoring, PDB code 2AQU.

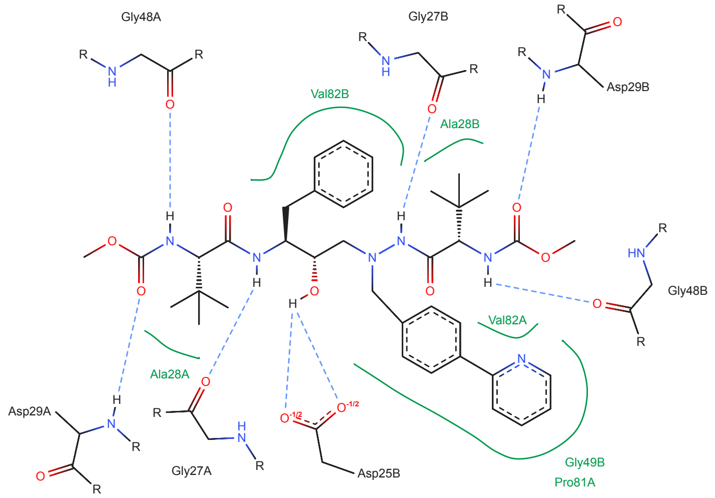

Supplementary Figure 35: Target 02-HIV-PR, 2D structures of the ligands in the series and their binding free energies derived from the experiment.

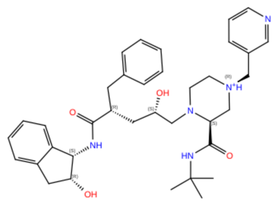

1HSG

ΔG_{bind} = -13.6 kcal/mol

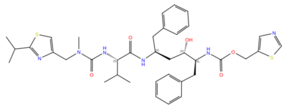

1HXW

ΔG_{bind} = -14.9 kcal/mol

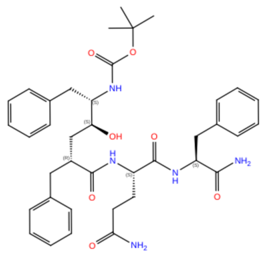

1IZH

ΔG_{bind} = -14.7 kcal/mol

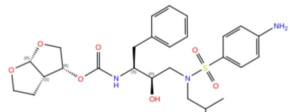

1T3R

ΔG_{bind} = -15.5 kcal/mol

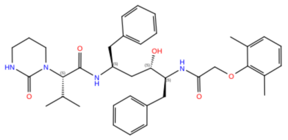

2Q54

ΔG_{bind} = -12.4 kcal/mol

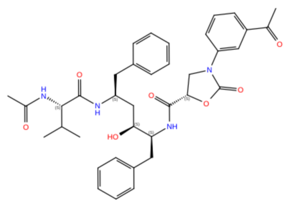

2Q55

ΔG_{bind} = -11.9 kcal/mol

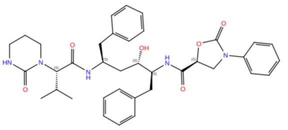

2Q5K

ΔG_{bind} = -14.7 kcal/mol

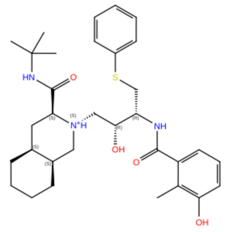

3EKX

ΔG_{bind} = -13.9 kcal/mol

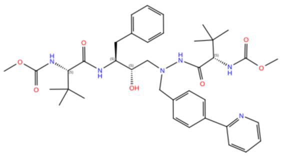

3EL1

ΔG_{bind} = -14.6 kcal/mol

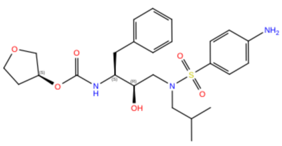

3NU3

ΔG_{bind} = -13.4 kcal/mol

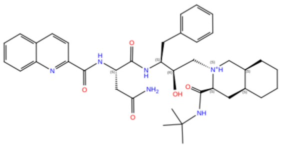

3OXC

ΔG_{bind} = -14.3 kcal/mol

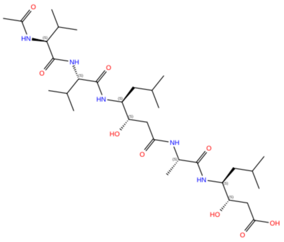

5HVP

ΔG_{bind} = -9.5 kcal/mol

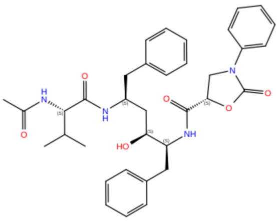

model13a

ΔG_{bind} = -12.5 kcal/mol

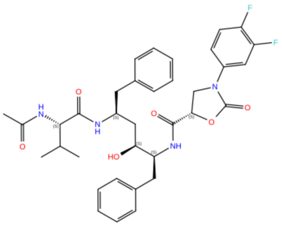

model13c

ΔG_{bind} = -11.9 kcal/mol

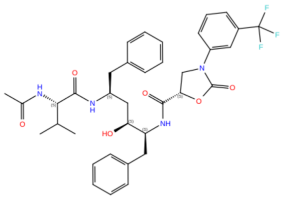

model13d

ΔG_{bind} = -11.8 kcal/mol

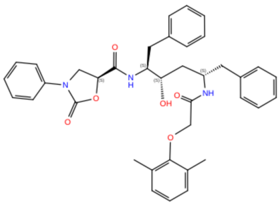

model16a

ΔG_{bind} = -11.1 kcal/mol

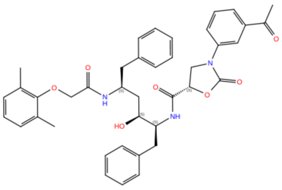

model16e

ΔG_{bind} = -10.2 kcal/mol

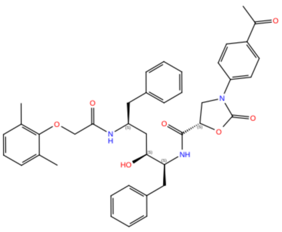

model16f

ΔG_{bind} = -10.2 kcal/mol

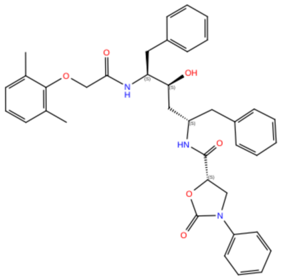

model22a

ΔG_{bind} = -11.2 kcal/mol

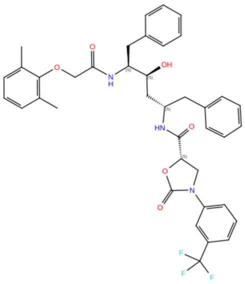

model22d

ΔG_{bind} = -10.5 kcal/mol

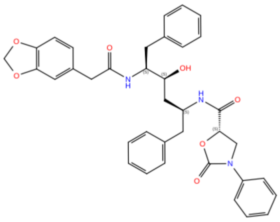

model23a

ΔG_{bind} = -8.5 kcal/mol

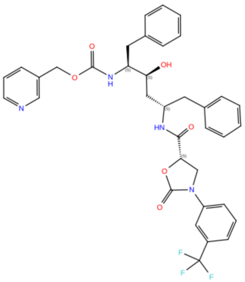

model25d

ΔG_{bind} = -10.1 kcal/mol

Supplementary Figure 36: Target 03-CK2, schematic drawing of the binding pose in the crystal selected for scoring, PDB code 3KXN.

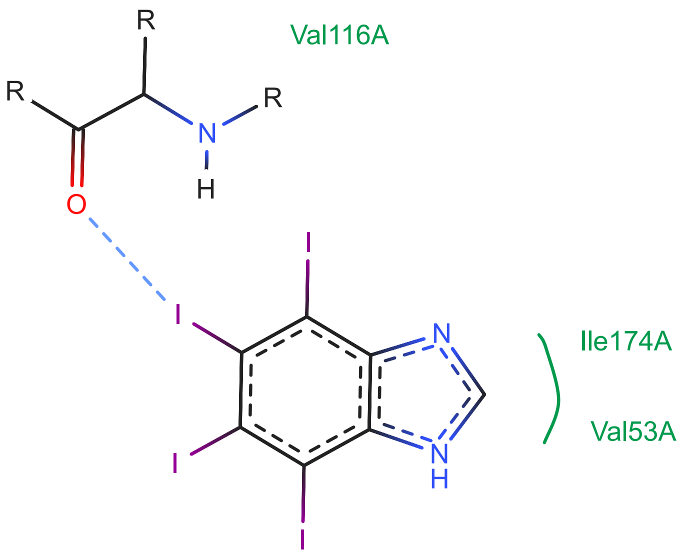

Supplementary Figure 37: Target 03-CK2, 2D structures of the ligands in the series and their binding free energies derived from the experiment.

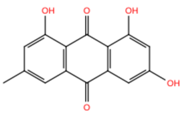

1F0Q

ΔG_{bind} = -7.9 kcal/mol

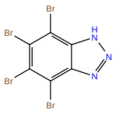

1J91

ΔG_{bind} = -8.8 kcal/mol

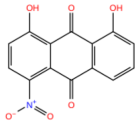

1M2P

ΔG_{bind} = -8.4 kcal/mol

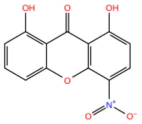

1M2Q

ΔG_{bind} = -8.4 kcal/mol

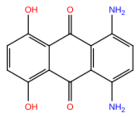

1M2R

ΔG_{bind} = -8.9 kcal/mol

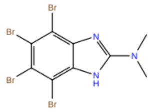

1ZOE

ΔG_{bind} = -10.1 kcal/mol

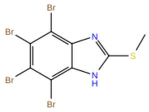

1ZOG

ΔG_{bind} = -9.8 kcal/mol

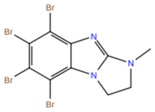

1ZOH

ΔG_{bind} = -9.6 kcal/mol

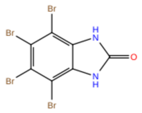

2OXD

ΔG_{bind} = -9.4 kcal/mol

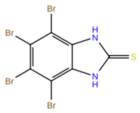

2OXX

ΔG_{bind} = -9.2 kcal/mol

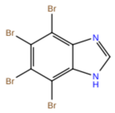

2OXY

ΔG_{bind} = -9.0 kcal/mol

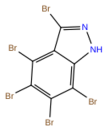

3KXG

ΔG_{bind} = -10.0 kcal/mol

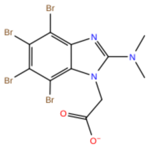

3KXH

ΔG_{bind} = -9.1 kcal/mol

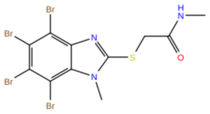

3KXM

ΔG_{bind} = -9.0 kcal/mol

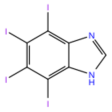

3KXN

ΔG_{bind} = -10.5 kcal/mol

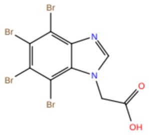

3PVG

ΔG_{bind} = -8.1 kcal/mol

Supplementary Figure 38: Target 04-AR, schematic drawing of the binding pose in the crystal selected for scoring, PDB code 4XZH.

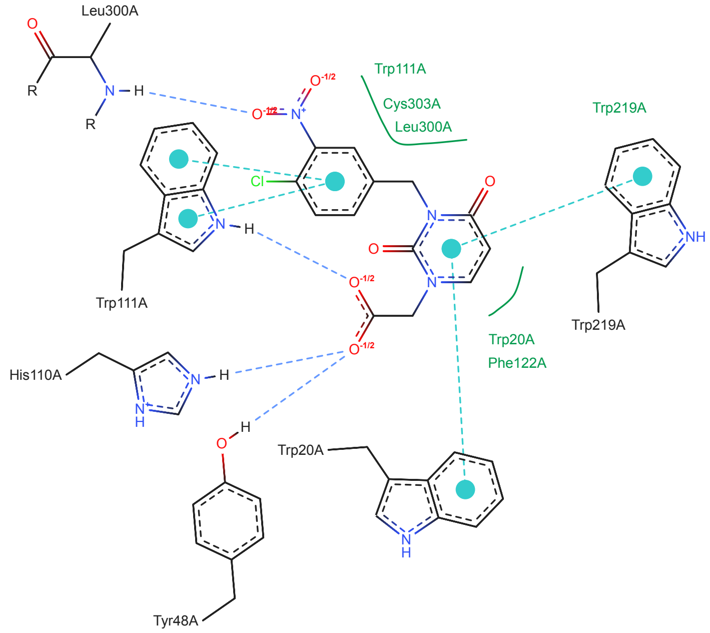

Supplementary Figure 39: Target 04-AR, 2D structures of the ligands in the series and their binding free energies derived from the experiment.

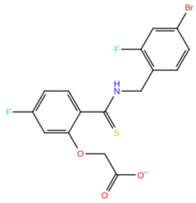

1US0

ΔG_{bind} = -9.6 kcal/mol

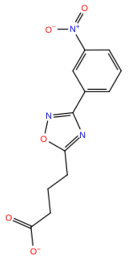

2IKG

ΔG_{bind} = -8.5 kcal/mol

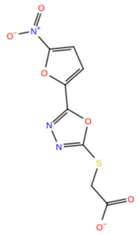

2IKH

ΔG_{bind} = -7.5 kcal/mol

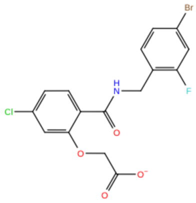

2IKI

ΔG_{bind} = -10.2 kcal/mol

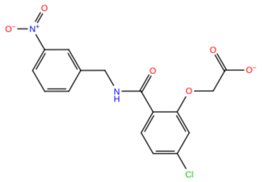

2IKJ

ΔG_{bind} = -10.1 kcal/mol

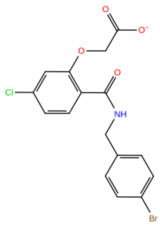

4LAU

ΔG_{bind} = -9.9 kcal/mol

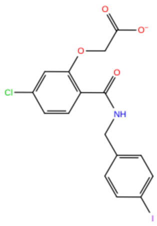

4LAZ

ΔG_{bind} = -9.4 kcal/mol

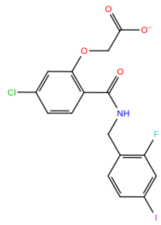

4LB3

ΔG_{bind} = -10.7 kcal/mol

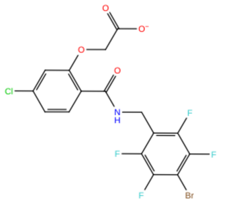

4LB4

ΔG_{bind} = -10.5 kcal/mol

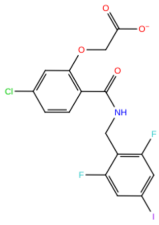

4LBR

ΔG_{bind} = -9.6 kcal/mol

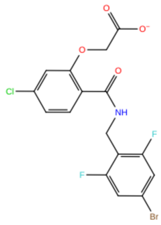

4LBS

ΔG_{bind} = -9.6 kcal/mol

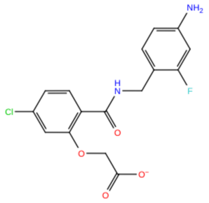

4QXI

ΔG_{bind} = -9.6 kcal/mol

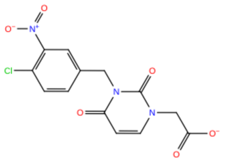

4XZH

ΔG_{bind} = -9.7 kcal/mol

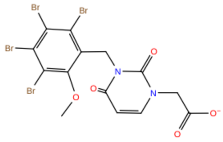

4XZI

ΔG_{bind} = -6.9 kcal/mol

Supplementary Figure 40: Target 05-Cath-D, schematic drawing of the binding pose in the crystal selected for scoring, PDB code 6QCB.

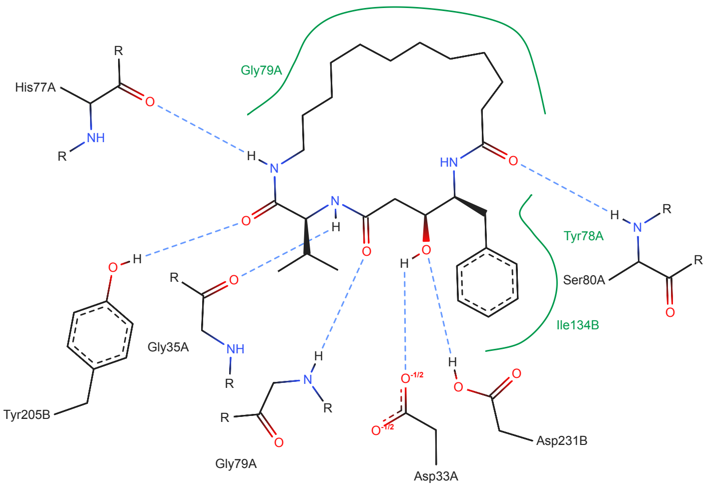

Supplementary Figure 41: Target 05-Cath-D, 2D structures of the ligands in the series and their binding free energies derived from the experiment.

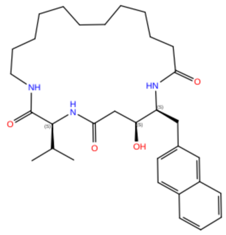

6QBG

ΔG_{bind} = -13.1 kcal/mol

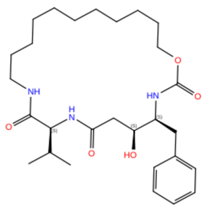

6QBH

ΔG_{bind} = -12.2 kcal/mol

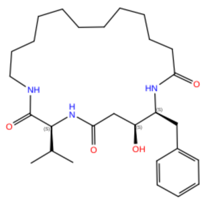

6QCB

ΔG_{bind} = -11.7 kcal/mol

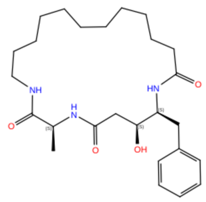

model08

ΔG_{bind} = -10.2 kcal/mol

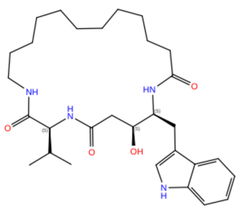

model22

ΔG_{bind} = -9.6 kcal/mol

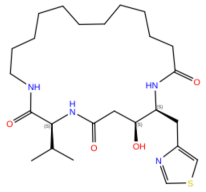

model24

ΔG_{bind} = -9.0 kcal/mol

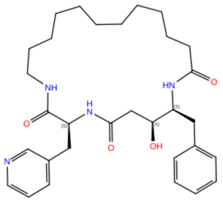

model25

ΔG_{bind} = -12.7 kcal/mol

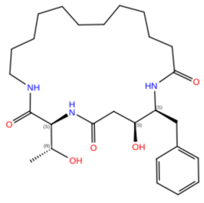

model31

ΔG_{bind} = -9.1 kcal/mol

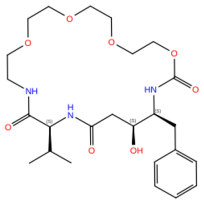

model35

ΔG_{bind} = -8.7 kcal/mol

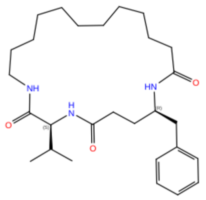

model36

ΔG_{bind} = -8.2 kcal/mol

Supplementary Figure 42: Target 06-BACE1, schematic drawing of the binding pose in the crystal selected for scoring, PDB code 5QCZ.

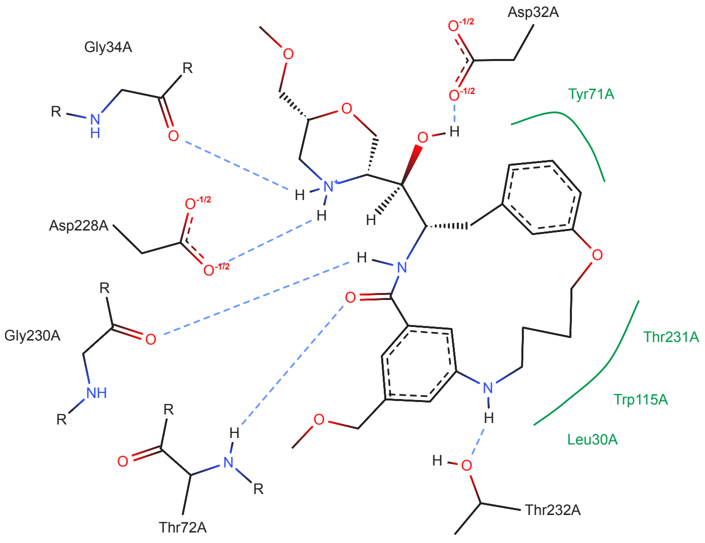

Supplementary Figure 43: Target 06-BACE1, 2D structures of the ligands in the series and their binding free energies derived from the experiment.

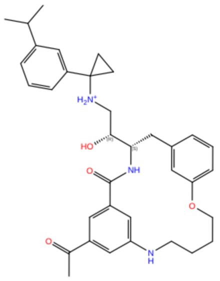

5QCO

ΔG_{bind} = -11.5 kcal/mol

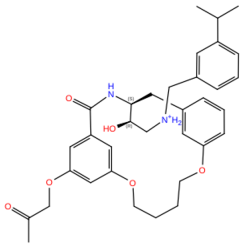

5QCP

ΔG_{bind} = -10.7 kcal/mol

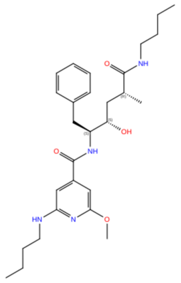

5QCR

ΔG_{bind} = -8.8 kcal/mol

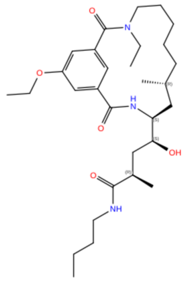

5QCT

ΔG_{bind} = -11.1 kcal/mol

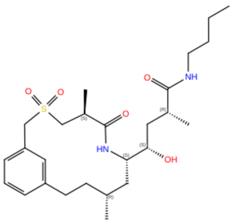

5QCU

ΔG_{bind} = -9.5 kcal/mol

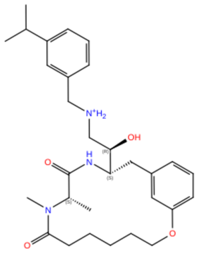

5QCV

ΔG_{bind} = -10.7 kcal/mol

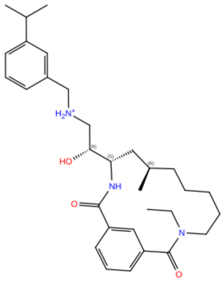

5QCX

ΔG_{bind} = -11.0 kcal/mol

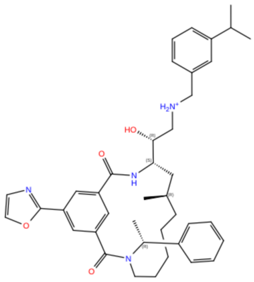

5QCY

ΔG_{bind} = -12.4 kcal/mol

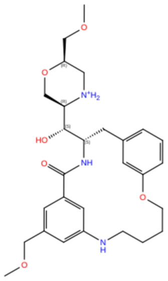

5QCZ

ΔG_{bind} = -7.4 kcal/mol

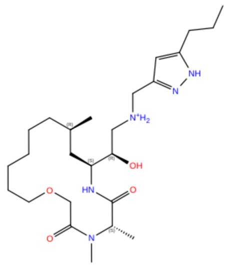

5QD0

ΔG_{bind} = -9.2 kcal/mol

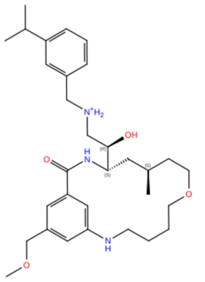

5QD1

ΔG_{bind} = -9.0 kcal/mol

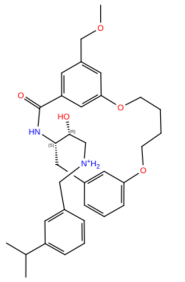

5QD2

ΔG_{bind} = -10.2 kcal/mol

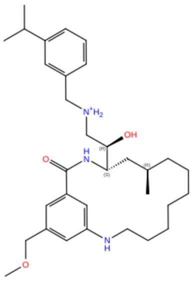

5QD3

ΔG_{bind} = -10.1 kcal/mol

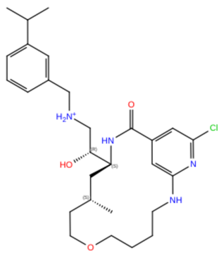

5QD5

ΔG_{bind} = -10.7 kcal/mol

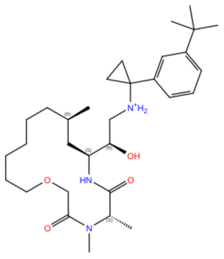

5QD9

ΔG_{bind} = -11.6 kcal/mol

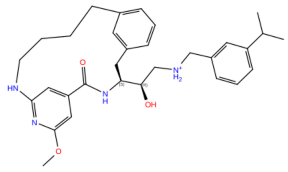

5QDA

ΔG_{bind} = -11.5 kcal/mol

Supplementary Figure 44: Target 07-JAK1, schematic drawing of the binding pose in the crystal selected for scoring, PDB code 4IVD.

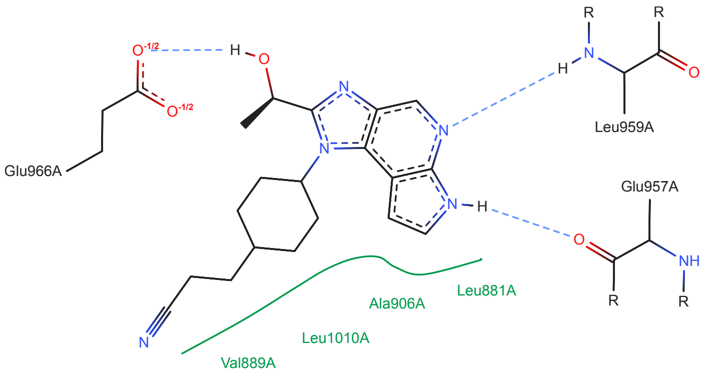

Supplementary Figure 45: Target 07-JAK1, 2D structures of the ligands in the series and their binding free energies derived from the experiment.

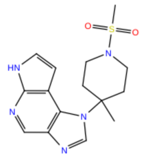

4E4L

ΔG_{bind} = -12.0 kcal/mol

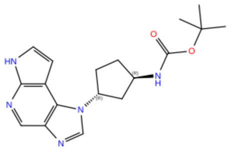

4E4N

ΔG_{bind} = -12.4 kcal/mol

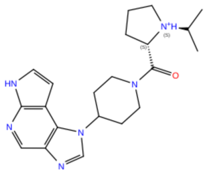

4E5W

ΔG_{bind} = -10.5 kcal/mol

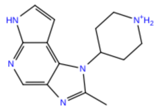

4EHZ

ΔG_{bind} = -11.0 kcal/mol

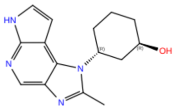

4EI4

ΔG_{bind} = -12.0 kcal/mol

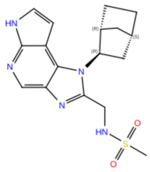

4FK6

ΔG_{bind} = -13.3 kcal/mol

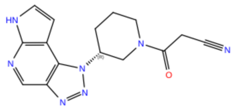

4I5C

ΔG_{bind} = -12.1 kcal/mol

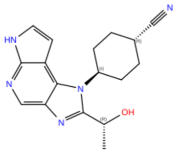

4IVB

ΔG_{bind} = -12.0 kcal/mol

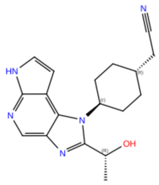

4IVC

ΔG_{bind} = -13.7 kcal/mol

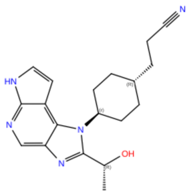

4IVD

ΔG_{bind} = -13.1 kcal/mol

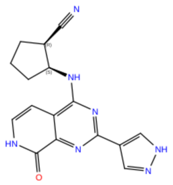

4K6Z

ΔG_{bind} = -11.9 kcal/mol

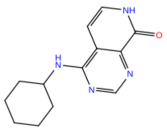

4K77

ΔG_{bind} = -9.1 kcal/mol

Supplementary Figure 46: Target 08-Trypsin, schematic drawing of the binding pose in the crystal selected for scoring, PDB code 1K1I.

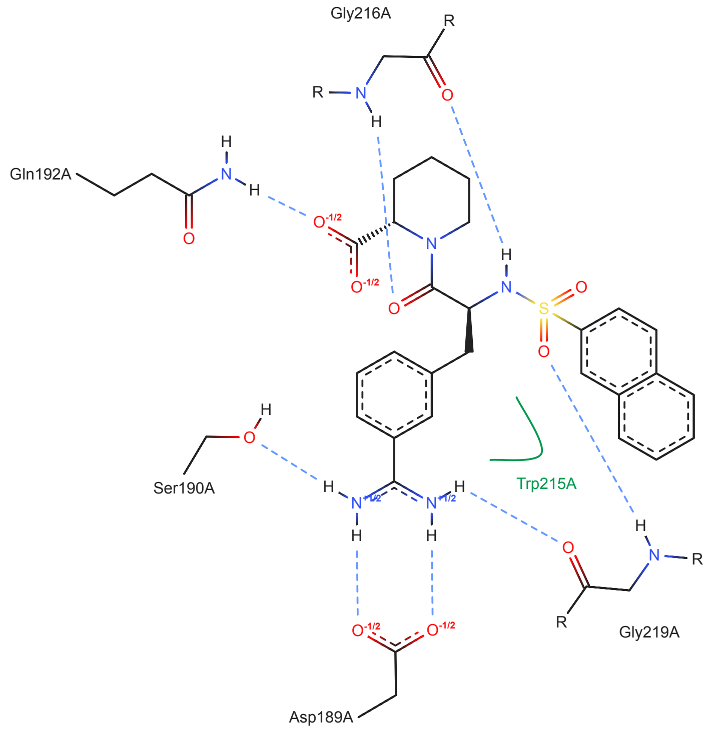

Supplementary Figure 47: Target 08-Trypsin, 2D structures of the ligands in the series and their binding free energies derived from the experiment.

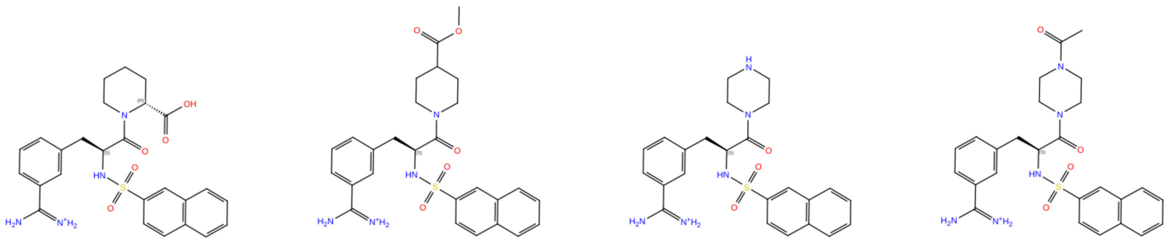

**1K1I**	1K1J	1K1L	1K1M
ΔG_{bind} = -9.0 kcal/mol	ΔG_{bind} = -10.3 kcal/mol	ΔG_{bind} = -9.4 kcal/mol	ΔG_{bind} = -10.1 kcal/mol

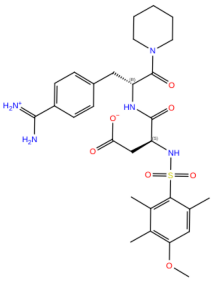

1K1N

ΔG_{bind} = -9.3 kcal/mol

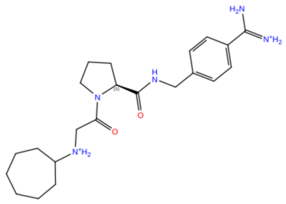

2ZHD

ΔG_{bind} = -8.9 kcal/mol

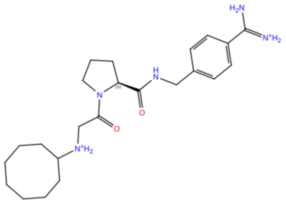

2ZQ2

ΔG_{bind} = -9.0 kcal/mol

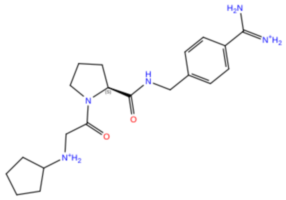

3LJJ

ΔG_{bind} = -8.9 kcal/mol

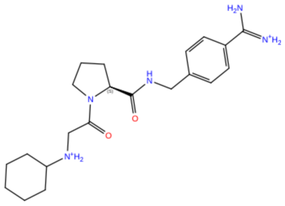

3LJO

ΔG_{bind} = -9.0 kcal/mol

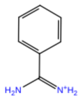

5MNG

ΔG_{bind} = -6.4 kcal/mol

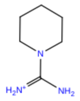

5MO2

ΔG_{bind} = -5.2 kcal/mol

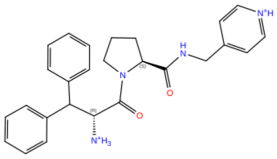

6SY3

ΔG_{bind} = -4.3 kcal/mol

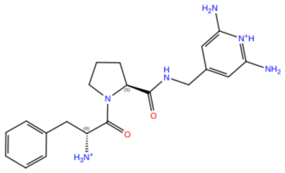

6T0M

ΔG_{bind} = -5.7 kcal/mol

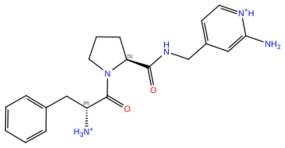

6T0P

ΔG_{bind} = -6.0 kcal/mol

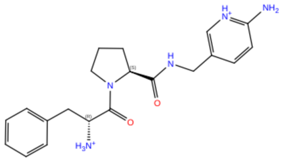

6T5W

ΔG_{bind} = -6.0 kcal/mol

Supplementary Figure 48: Target 09-CDK2, schematic drawing of the binding pose in the crystal selected for scoring, PDB code 3R9D.

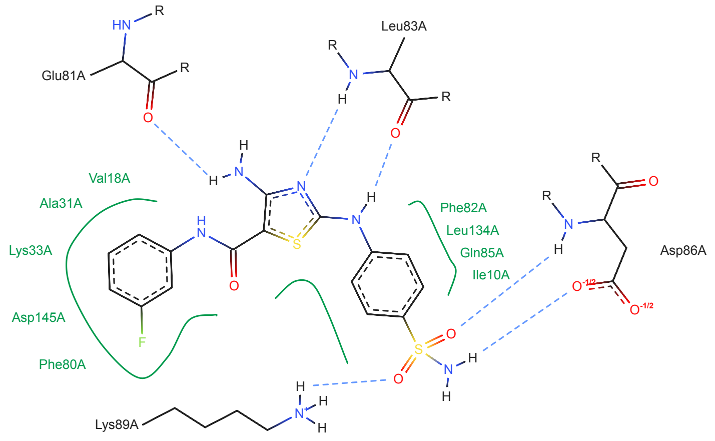

Supplementary Figure 49: Target 09-CDK2, 2D structures of the ligands in the series and their binding free energies derived from the experiment.

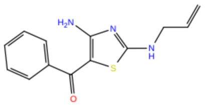

3QQK

ΔG_{bind} = -7.0 kcal/mol

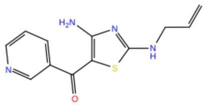

3QTQ

ΔG_{bind} = -8.0 kcal/mol

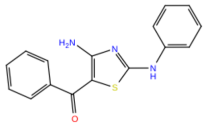

3QTR

ΔG_{bind} = -8.7 kcal/mol

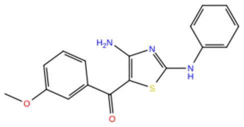

3QTS

ΔG_{bind} = -8.0 kcal/mol

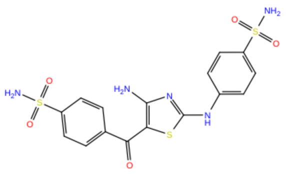

3QTU

ΔG_{bind} = -10.2 kcal/mol

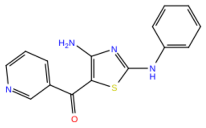

3QTW

ΔG_{bind} = -8.9 kcal/mol

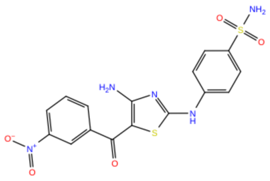

3QTX

ΔG_{bind} = -10.2 kcal/mol

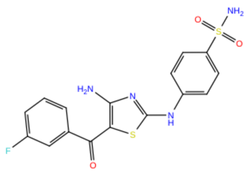

3QTZ

ΔG_{bind} = -10.4 kcal/mol

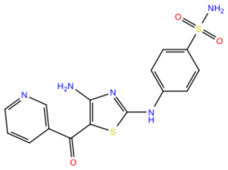

3QU0

ΔG_{bind} = -11.0 kcal/mol

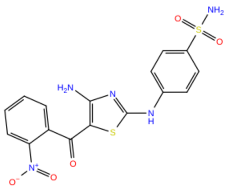

3QXP

ΔG_{bind} = -11.0 kcal/mol

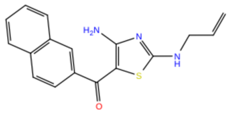

3R8U

ΔG_{bind} = -7.7 kcal/mol

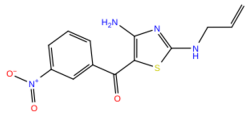

3R8V

ΔG_{bind} = -8.0 kcal/mol

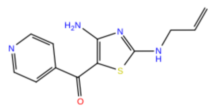

3R8Z

ΔG_{bind} = -6.3 kcal/mol

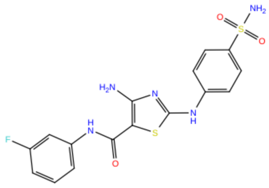

3R9D

ΔG_{bind} = -6.1 kcal/mol

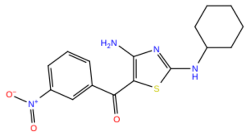

3R9N

ΔG_{bind} = -8.4 kcal/mol

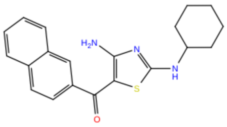

3RAH

ΔG_{bind} = -7.2 kcal/mol

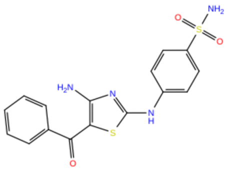

3RAK

ΔG_{bind} = -9.9 kcal/mol

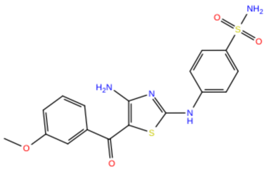

3RAL

ΔG_{bind} = -10.0 kcal/mol

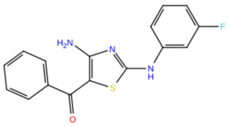

3RJC

ΔG_{bind} = -7.6 kcal/mol

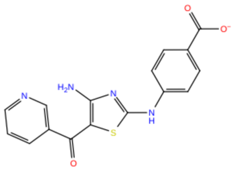

3RK5

ΔG_{bind} = -7.8 kcal/mol

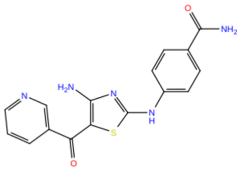

3RK7

ΔG_{bind} = -9.6 kcal/mol

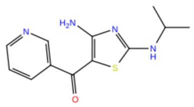

3RK9

ΔG_{bind} = -9.0 kcal/mol

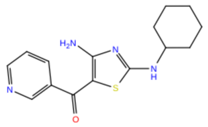

3RKB

ΔG_{bind} = -9.2 kcal/mol

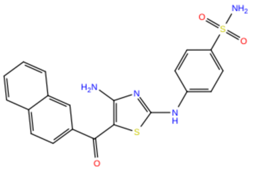

3RMF

ΔG_{bind} = -6.0 kcal/mol

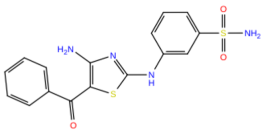

3RNI

ΔG_{bind} = -7.5 kcal/mol

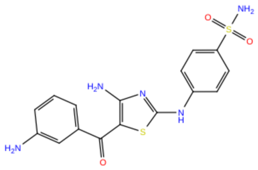

3RPV

ΔG_{bind} = -10.2 kcal/mol

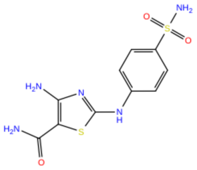

3RPY

ΔG_{bind} = -7.7 kcal/mol

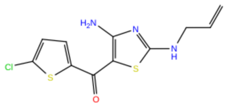

3S00

ΔG_{bind} = -7.2 kcal/mol

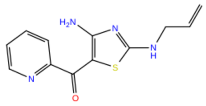

3S0O

ΔG_{bind} = -6.6 kcal/mol

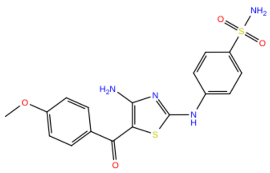

3S1H

ΔG_{bind} = -7.0 kcal/mol

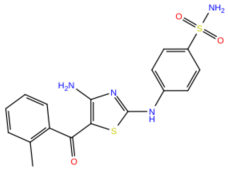

3SQQ

ΔG_{bind} = -7.7 kcal/mol

Supplementary Figure 50: Target 10-MMP12, schematic drawing of the binding pose in the crystal selected for scoring, PDB code 3EHY.

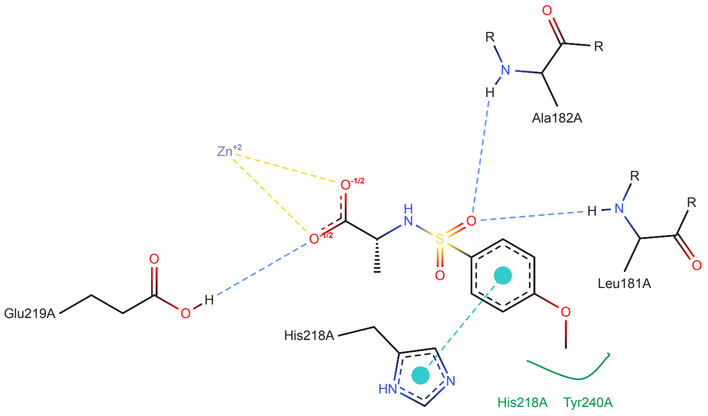

Supplementary Figure 51: Target 10-MMP12, 2D structures of the ligands in the series and their binding free energies derived from the experiment.

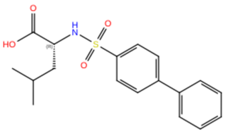

3EHX

ΔG_{bind} = -10.4 kcal/mol

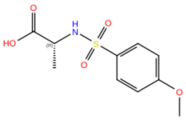

3EHY

ΔG_{bind} = -8.0 kcal/mol

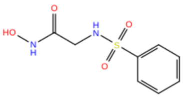

3F15

ΔG_{bind} = -11.1 kcal/mol

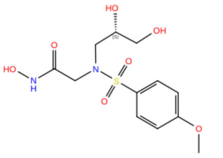

3F16

ΔG_{bind} = -11.2 kcal/mol

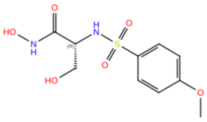

3F17

ΔG_{bind} = -11.8 kcal/mol

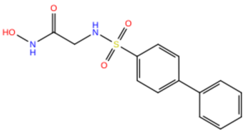

3F18

ΔG_{bind} = -10.1 kcal/mol

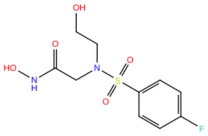

3F19

ΔG_{bind} = -9.8 kcal/mol

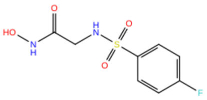

3F1A

ΔG_{bind} = -9.9 kcal/mol

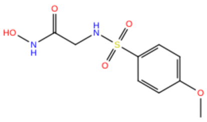

3LK8

ΔG_{bind} = -10.5 kcal/mol

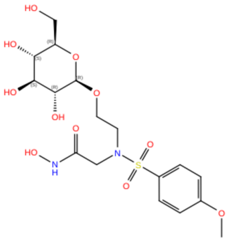

3N2U

ΔG_{bind} = -10.7 kcal/mol

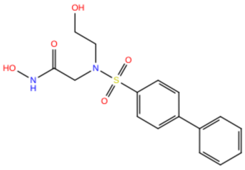

3N2V

ΔG_{bind} = -10.2 kcal/mol

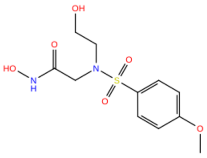

3NX7

ΔG_{bind} = -11.1 kcal/mol

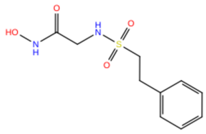

3RTS

ΔG_{bind} = -10.4 kcal/mol

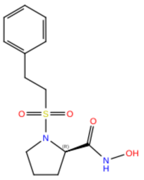

3RTT

ΔG_{bind} = -10.6 kcal/mol

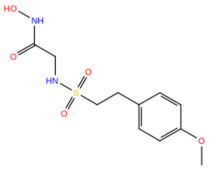

4GUY

ΔG_{bind} = -10.9 kcal/mol

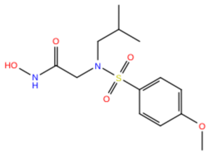

5LAB

ΔG_{bind} = -11.4 kcal/mol

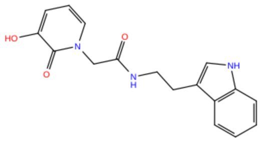

6RD0

ΔG_{bind} = -6.5 kcal/mol

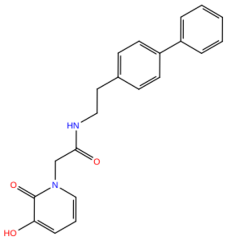

6RLY

ΔG_{bind} = -6.5 kcal/mol

S12) References

1 Pecina, A. *et al.* Ranking Power of the SQM/COSMO Scoring Function on Carbonic Anhydrase II-Inhibitor Complexes. *ChemPhysChem* **19**, 873-879, doi:10.1002/cphc.201701104 (2018).

2 Weber, J. *et al.* Unusual binding mode of an HIV-1 protease inhibitor explains its potency against multi-drug-resistant virus strains. *Journal of Molecular Biology* **324**, 739-754, doi:10.1016/S0022-2836(02)01139-7 (2002).

3 Fanfrlik, J. *et al.* A reliable docking/scoring scheme based on the semiempirical quantum mechanical PM6-DH2 method accurately covering dispersion and H-bonding: HIV-1 protease with 22 ligands. *J Phys Chem B* **114**, 12666-12678, doi:10.1021/jp1032965 (2010).

4 Reddy, G. S. *et al.* Design and synthesis of HIV-1 protease inhibitors incorporating oxazolidinones as P2/P2' ligands in pseudosymmetric dipeptide isosteres. *J Med Chem* **50**, 4316-4328, doi:10.1021/jm070284z (2007).

5 Kramer, C., Kalliokoski, T., Gedeck, P. & Vulpetti, A. The experimental uncertainty of heterogeneous public K(i) data. *J Med Chem* **55**, 5165-5173, doi:10.1021/jm300131x (2012).

6 De Moliner, E. *et al.* Inhibition of protein kinase CK2 by anthraquinone-related compounds. A structural insight. *J Biol Chem* **278**, 1831-1836, doi:10.1074/jbc.M209367200 (2003).

7 Battistutta, R. *et al.* Inspecting the structure-activity relationship of protein kinase CK2 inhibitors derived from tetrabromo-benzimidazole. *Chem Biol* **12**, 1211-1219, doi:10.1016/j.chembiol.2005.08.015 (2005).

8 Sarno, S. *et al.* ATP site-directed inhibitors of protein kinase CK2: an update. *Curr Top Med Chem* **11**, 1340-1351, doi:10.2174/156802611795589638 (2011).

9 Battistutta, R. *et al.* The replacement of ATP by the competitive inhibitor emodin induces conformational modifications in the catalytic site of protein kinase CK2. *Journal of Biological Chemistry* **275**, 29618-29622, doi:DOI 10.1074/jbc.M004257200 (2000).

10 Battistutta, R., De Moliner, E., Sarno, S., Zanotti, G. & Pinna, L. A. Structural features underlying selective inhibition of protein kinase CK2 by ATP site-directed tetrabromo-2-benzotriazole. *Protein Sci* **10**, 2200-2206, doi:DOI 10.1110/ps.19601 (2001).

11 Battistutta, R. *et al.* The ATP-binding site of protein kinase CK2 holds a positive electrostatic area and conserved water molecules. *Chembiochem* **8**, 1804-1809, doi:10.1002/cbic.200700307 (2007).

12 Steuber, H., Heine, A. & Klebe, G. Structural and thermodynamic study on aldose reductase: nitro-substituted inhibitors with strong enthalpic binding contribution. *J Mol Biol* **368**, 618-638, doi:10.1016/j.jmb.2006.12.004 (2007).

13 Koch, C., Heine, A. & Klebe, G. Tracing the detail: how mutations affect binding modes and thermodynamic signatures of closely related aldose reductase inhibitors. *J Mol Biol* **406**, 700-712, doi:10.1016/j.jmb.2010.11.058 (2011).

14 Fanfrlík, J. *et al.* The Effect of Halogen-to-Hydrogen Bond Substitution on Human Aldose Reductase Inhibition. *ACS Chem Biol* **10**, 1637-1642, doi:10.1021/acschembio.5b00151 (2015).

15 Fanfrlík, J. *et al.* Modulation of aldose reductase inhibition by halogen bond tuning. *ACS Chem Biol* **8**, 2484-2492, doi:10.1021/cb400526n (2013).

16 Ruiz, F. X. *et al.* Structural Determinants of the Selectivity of 3-Benzyluracil-1-acetic Acids toward Human Enzymes Aldose Reductase and AKR1B10. *Chemmedchem* **10**, 1989-2003, doi:10.1002/cmdc.201500393 (2015).

17 Houštecká, R. *et al.* Biomimetic Macrocyclic Inhibitors of Human Cathepsin D: Structure-Activity Relationship and Binding Mode Analysis. *J Med Chem* **63**, 1576-1596, doi:10.1021/acs.jmedchem.9b01351 (2020).

18 Hanessian, S. *et al.* Structure-based design and synthesis of macroheterocyclic peptidomimetic inhibitors of the aspartic protease beta-site amyloid precursor protein cleaving enzyme (BACE). *Journal of Medicinal Chemistry* **49**, 4544-4567, doi:10.1021/jm060154a (2006).

19 Labadie, S. *et al.* Structure-based discovery of C-2 substituted imidazo-pyrrolopyridine JAK1 inhibitors with improved selectivity over JAK2. *Bioorg Med Chem Lett* **22**, 7627-7633, doi:10.1016/j.bmcl.2012.10.008 (2012).
20 Zak, M. *et al.* Discovery and optimization of potent, selective, and orally bioavailable Jak1 inhibitors. *Abstr Pap Am Chem S* **243** (2012).
21 Kulagowski, J. J. *et al.* Identification of imidazo-pyrrolopyridines as novel and potent JAK1 inhibitors. *J Med Chem* **55**, 5901-5921, doi:10.1021/jm300438j (2012).
22 Hurley, C. A. *et al.* Novel triazolo-pyrrolopyridines as inhibitors of Janus kinase 1. *Bioorg Med Chem Lett* **23**, 3592-3598, doi:10.1016/j.bmcl.2013.04.018 (2013).
23 Zak, M. *et al.* Identification of C-2 hydroxyethyl imidazopyrrolopyridines as potent JAK1 inhibitors with favorable physicochemical properties and high selectivity over JAK2. *J Med Chem* **56**, 4764-4785, doi:10.1021/jm4004895 (2013).
24 Labadie, S. S. *et al.* Design and evaluation of novel 8-oxo-pyridopyrimidine Jak1/2 inhibitors. *Abstr Pap Am Chem S* **246** (2013).
25 Dullweber, F., Stubbs, M. T., Musil, D., Sturzebecher, J. & Klebe, G. Factorising ligand affinity: a combined thermodynamic and crystallographic study of trypsin and thrombin inhibition. *J Mol Biol* **313**, 593-614, doi:10.1006/jmbi.2001.5062 (2001).
26 Brandt, T. *et al.* Congeneric but still distinct: how closely related trypsin ligands exhibit different thermodynamic and structural properties. *J Mol Biol* **405**, 1170-1187, doi:10.1016/j.jmb.2010.11.038 (2011).
27 Schiebel, J. *et al.* Charges Shift Protonation: Neutron Diffraction Reveals that Aniline and 2-Aminopyridine Become Protonated Upon Binding to Trypsin. *Angew Chem Int Edit* **56**, 4887-4890, doi:10.1002/anie.201701038 (2017).
28 Ngo, K. *et al.* Protein-Induced Change in Ligand Protonation during Trypsin and Thrombin Binding: Hint on Differences in Selectivity Determinants of Both Proteins? *J Med Chem* **63**, 3274-3289, doi:10.1021/acs.jmedchem.9b02061 (2020).
29 Schiebel, J. *et al.* Intriguing role of water in protein-ligand binding studied by neutron crystallography on trypsin complexes. *Nat Commun* **9**, 3559, doi:10.1038/s41467-018-05769-2 (2018).
30 Schmidt, A., Jelsch, C., Ostergaard, P., Rypniewski, W. & Lamzin, V. S. Trypsin revisited: crystallography AT (SUB) atomic resolution and quantum chemistry revealing details of catalysis. *J Biol Chem* **278**, 43357-43362, doi:10.1074/jbc.M306944200 (2003).
31 Schonbrunn, E. *et al.* Development of highly potent and selective diaminothiazole inhibitors of cyclin-dependent kinases. *J Med Chem* **56**, 3768-3782, doi:10.1021/jm301234k (2013).
32 Bertini, I. *et al.* Exploring the subtleties of drug-receptor interactions: the case of matrix metalloproteinases. *J Am Chem Soc* **129**, 2466-2475, doi:10.1021/ja065156z (2007).
33 Dragoni, E. *et al.* Biotin-tagged probes for MMP expression and activation: design, synthesis, and binding properties. *Bioconjug Chem* **20**, 719-727, doi:10.1021/bc8003827 (2009).
34 Attolino, E. *et al.* Structure-based approach to nanomolar, water soluble matrix metalloproteinases inhibitors (MMPIs). *Eur J Med Chem* **45**, 5919-5925, doi:10.1016/j.ejmech.2010.09.057 (2010).
