# Supplementary material for: SQM2.20: Semiempirical quantum-mechanical scoring function yields DFT-quality protein–ligand binding affinity predictions in minutes
Source: Nat Commun. 2024 Feb 6;15:1127. doi: 10.1038/s41467-024-45431-8 (PMC10847445; doi:10.1038/s41467-024-45431-8)
Supplement: Supplementary file 2 — Reporting Summary [file 41467_2024_45431_MOESM2_ESM.pdf]

nature portfolio

Corresponding author(s): Jan Řezáč

Last updated by author(s): 2024/11/01

Reporting Summary

Nature Portfolio wishes to improve the reproducibility of the work that we publish. This form provides structure for consistency and transparency in reporting. For further information on Nature Portfolio policies, see our Editorial Policies and the Editorial Policy Checklist.

Statistics

For all statistical analyses, confirm that the following items are present in the figure legend, table legend, main text, or Methods section.

n/a	Confirmed	
☐	☒	The exact sample size (*n*) for each experimental group/condition, given as a discrete number and unit of measurement
☒	☐	A statement on whether measurements were taken from distinct samples or whether the same sample was measured repeatedly
☒	☐	The statistical test(s) used AND whether they are one- or two-sided<br>*Only common tests should be described solely by name; describe more complex techniques in the Methods section.*
☒	☐	A description of all covariates tested
☒	☐	A description of any assumptions or corrections, such as tests of normality and adjustment for multiple comparisons
☐	☒	A full description of the statistical parameters including central tendency (e.g. means) or other basic estimates (e.g. regression coefficient) AND variation (e.g. standard deviation) or associated estimates of uncertainty (e.g. confidence intervals)
☒	☐	For null hypothesis testing, the test statistic (e.g. *F*, *t*, *r*) with confidence intervals, effect sizes, degrees of freedom and *P* value noted<br>*Give P values as exact values whenever suitable.*
☒	☐	For Bayesian analysis, information on the choice of priors and Markov chain Monte Carlo settings
☒	☐	For hierarchical and complex designs, identification of the appropriate level for tests and full reporting of outcomes
☐	☒	Estimates of effect sizes (e.g. Cohen's *d*, Pearson's *r*), indicating how they were calculated

Our web collection on statistics for biologists contains articles on many of the points above.

Software and code

Policy information about availability of computer code

Data collection	The software central to this research is open source and well-documented (MOPAC, CUBY4, AmberTools) and cited in the paper. The DFT calculations were performed in ORCA v.5.0.3. We used 2 commercial programms for scoring (CCDC GOLD Suite v.2022.1.0 and GLIDE in Schrodinger software (v.2022-1). Nine open source academic scoring functions were used, i.e., Plants v.1.2, X-Score v1.2, AutoDock v.4.2.6, Smina fork of Autodock, dVinaRF20, NNScore2.0, RF-score-VS, Pafnucy. For system preparation, we used the following software and codes: Hydrogens and missing heavy atoms were added to the protein using the Leap tool of AMBER 20 suite; Hydrogens were added to the ligands by using the software Obabel v. 2.3 (http://openbabel.org); pKa calculations were done in Stardrop v. 7.0 (https://optibrium.com/stardrop); the geometry optimizations were performed in Cuby4 (http://cuby4.molecular.cz), which also provided the interface implementing the SQM/MM scheme. The software central to the SQM2.20 scoring i.e., MOPAC2016 (http://openmopac.net), Cuby4 and AmberTools (https://ambermd.org), is open source and well-documented, and properly cited in the paper. The DFT-score calculations were performed in ORCA v.5.0.3. Two commercial scoring packages (CCDC GOLD Suite v.2022.1.0 and GLIDE module (v. 9.4.141, mmshare v. 5.7.141) in Schrodinger software (v.2022-1) and nine open source academic packages, i.e., Plants v.1.2, X-Score v1.2, AutoDock v.4.2.6, Smina fork of Autodock, dVinaRF20, NNScore 2.0, RF-score-VS version 2, Pafnucy (http://gitlab.com/cheminfIBB/pafnucy), were used for re-scoring.
Data analysis	Data were collected by standard command-line tools, processed and analysed in the spreadsheet and plotted using Gnuplot.

For manuscripts utilizing custom algorithms or software that are central to the research but not yet described in published literature, software must be made available to editors and reviewers. We strongly encourage code deposition in a community repository (e.g. GitHub). See the Nature Portfolio guidelines for submitting code & software for further information.

Data

Policy information about availability of data

All manuscripts must include a data availability statement. This statement should provide the following information, where applicable:

- Accession codes, unique identifiers, or web links for publicly available datasets
- A description of any restrictions on data availability
- For clinical datasets or third party data, please ensure that the statement adheres to our policy

The prepared and optimized structures of the protein-ligand complexes, the PL-REX data set, as well as other structures used in the calculations reported in the paper, and the resulting scores generated in this study have been deposited in a GitHub repository https://github.com/Honza-R/PL-REX and are also archived at Zenodo with DOI 10.5281/zenodo.8182922.14 The crystal structures used in this work are available in the RSCB Protein Data Bank (https://www.rcsb.org/) under the codes listed in the paper. The source data for the tables and plots presented in the paper and in the Supplementary Information are also provided along with the paper as the Source Data file.

Research involving human participants, their data, or biological material

Policy information about studies with human participants or human data. See also policy information about sex, gender (identity/presentation), and sexual orientation and race, ethnicity and racism.

Reporting on sex and gender	N/A
Reporting on race, ethnicity, or other socially relevant groupings	N/A
Population characteristics	N/A
Recruitment	N/A
Ethics oversight	N/A

Note that full information on the approval of the study protocol must also be provided in the manuscript.

Field-specific reporting

Please select the one below that is the best fit for your research. If you are not sure, read the appropriate sections before making your selection.

☒ Life sciences ☐ Behavioural & social sciences ☐ Ecological, evolutionary & environmental sciences

For a reference copy of the document with all sections, see nature.com/documents/nr-reporting-summary-flat.pdf

Life sciences study design

All studies must disclose on these points even when the disclosure is negative.

Sample size	164 data points corresponding to protein-ligand complexes from literature matching criteria described in the manuscript.
Data exclusions	No data were excluded from the analyses based on any pre-established criteria. In the DFT scoring, iodine-containing ligands of 03-CK2 and 04-AR datasets were excluded from final statistics because these calculations did not converged. None of GOLD scoring functions were able to score the 3KXG complex of 03-CK2 series that was therefore not included in the final statistics. All these exceptions are clearly mentioned in the paper.
Replication	N/A
Randomization	N/A
Blinding	N/A

Reporting for specific materials, systems and methods

We require information from authors about some types of materials, experimental systems and methods used in many studies. Here, indicate whether each material, system or method listed is relevant to your study. If you are not sure if a list item applies to your research, read the appropriate section before selecting a response.

Materials & experimental systems

n/a	Involved in the study	
☒	☐	Antibodies
☒	☐	Eukaryotic cell lines
☒	☐	Palaeontology and archaeology
☒	☐	Animals and other organisms
☒	☐	Clinical data
☒	☐	Dual use research of concern
☒	☐	Plants

Methods

n/a	Involved in the study	
☒	☐	ChIP-seq
☒	☐	Flow cytometry
☒	☐	MRI-based neuroimaging

Plants

Seed stocks	N/A
Novel plant genotypes	N/A
Authentication	N/A
